# Evaluating wildlife translocations using genomics: A bighorn sheep case study

**DOI:** 10.1002/ece3.6942

**Published:** 2020-11-21

**Authors:** Elizabeth P. Flesch, Tabitha A. Graves, Jennifer M. Thomson, Kelly M. Proffitt, P. J. White, Thomas R. Stephenson, Robert A. Garrott

**Affiliations:** ^1^ Fish and Wildlife Ecology and Management Program Ecology Department Montana State University Bozeman MT USA; ^2^ Northern Rocky Mountain Science Center U.S. Geological Survey West Glacier MT USA; ^3^ Animal and Range Sciences Department Montana State University Bozeman MT USA; ^4^ Montana Fish Wildlife and Parks Bozeman MT USA; ^5^ Yellowstone Center for Resources National Park Service Mammoth WY USA; ^6^ Sierra Nevada Bighorn Sheep Recovery Program California Department of Fish and Wildlife Bishop CA USA

**Keywords:** bighorn sheep, genomics, kinship, *Ovis canadensis*, population genetics, translocation

## Abstract

Wildlife restoration often involves translocation efforts to reintroduce species and supplement small, fragmented populations. We examined the genomic consequences of bighorn sheep (*Ovis canadensis*) translocations and population isolation to enhance understanding of evolutionary processes that affect population genetics and inform future restoration strategies. We conducted a population genomic analysis of 511 bighorn sheep from 17 areas, including native and reintroduced populations that received 0–10 translocations. Using the Illumina High Density Ovine array, we generated datasets of 6,155 to 33,289 single nucleotide polymorphisms and completed clustering, population tree, and kinship analyses. Our analyses determined that natural gene flow did not occur between most populations, including two pairs of native herds that had past connectivity. We synthesized genomic evidence across analyses to evaluate 24 different translocation events and detected eight successful reintroductions (i.e., lack of signal for recolonization from nearby populations) and five successful augmentations (i.e., reproductive success of translocated individuals) based on genetic similarity with the source populations. A single native population founded six of the reintroduced herds, suggesting that environmental conditions did not need to match for populations to persist following reintroduction. Augmentations consisting of 18–57 animals including males and females succeeded, whereas augmentations of two males did not result in a detectable genetic signature. Our results provide insight on genomic distinctiveness of native and reintroduced herds, information on the relative success of reintroduction and augmentation efforts and their associated attributes, and guidance to enhance genetic contribution of augmentations and reintroductions to aid in bighorn sheep restoration.

## Graphical abstract

We examined the genomic consequences of bighorn sheep (*Ovis canadensis*) translocations and population isolation to enhance understanding of evolutionary processes that affect population genetics and inform future restoration strategies. We conducted a population genomic analysis of 511 bighorn sheep from 17 areas, including native and reintroduced populations that received 0–10 translocations. Our results provide insight on genomic distinctiveness of native and reintroduced herds, information on the relative success of reintroduction and augmentation efforts and their associated attributes, and guidance to enhance genetic contribution of augmentations and reintroductions to aid in bighorn sheep restoration.

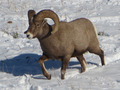

## 1 INTRODUCTION

Population restoration using translocation, which involves moving live individuals from one area to another, is an important tool for restoring biodiversity (IUCN/SSC, 2013; Weeks et al., 2011). Reintroduction starts a new population within formerly occupied landscapes, whereas augmentation adds individuals to an indigenous or reintroduced population (IUCN/SSC, 2013). The goal of augmenting a small, genetically isolated population is frequently to enhance viability by increasing the number of individuals in certain demographic groups or increasing genetic diversity to improve reproductive fitness, termed genetic rescue (IUCN/SSC, 2013; Tallmon et al., 2004).

Successful survival and breeding of translocated animals can depend on many factors, including environmental similarity between source and release areas in case of local adaptation, reproductive attributes of the species, habitat quality of the target area, distance from other conspecific populations, number of individuals moved, and management program duration (Bleich et al., 2018; Griffith et al., 1989; Groombridge et al., 2012; Magdalena Wolf et al., 1998). Managers can control some of the factors that influence survival and breeding of translocated animals to enhance the probability of translocation success. However, the relative survival and reproduction of translocated individuals in augmentations and reintroductions and the long‐term genetic effects of translocation efforts may vary widely by species and population of interest. Thus, it is beneficial to enhance understanding of the multi‐generational genetic effects of reintroduction and augmentation efforts in wild populations (Moraes et al., 2017; White et al., 2018).

While reintroductions can successfully establish a new wildlife population, previous studies have found that some populations thought to be the result of reintroduction efforts were in fact the result of recolonization (Kruckenhauser & Pinsker, 2004; Statham et al., 2012; Stewart et al., 2017). In this case, animals from nearby areas naturally dispersed and established a population in previously occupied terrain, meaning that costly reintroduction efforts may not have been necessary. Further, some studies have suggested that matching environmental attributes between the source population and the reintroduction area is important to establishment of translocated animals, whereas others have found that sourcing from a native, rather than reintroduced, population is more important (Malaney et al., 2015; Olson et al., 2013). Thus, evaluating the genetic source of populations thought to be reintroduced can provide insight into whether similar translocation efforts would be productive in other areas or whether enhancement of habitat connectivity would be more important to encourage recolonization (Stewart et al., 2017). Further, evaluating the genetic differences between populations that were the result of reintroduction events and their founding source can teach us about the many influences on the reintroduced population's evolution (Jamieson, 2011; White et al., 2018). For example, the literature frequently recommends large founder populations for reintroductions to represent the genetic profile of the source and minimize inbreeding (Jamieson & Lacy, 2012). However, the necessary founder size for reintroductions may vary widely by species, population, and reintroduction area of interest. Genetic evaluation of reintroduced populations can help address these uncertainties in reintroduction planning.

In addition, augmentation of existing populations may not result in genetic contribution to the target recipient population. Previous studies have found that the sex of animals moved and species mating strategy can influence whether translocated animals breed with the resident population (Miller et al., 2009; Mulder et al., 2017; Sigg et al., 2005). For example, no translocated males contributed to a resident desert tortoise (*Gopherus agassizii*) population; resident and translocated females only produced progeny with resident males (Mulder et al., 2017). Thus, determining what factors influence animal breeding following augmentation can help biologists avoid implementation of costly augmentation efforts that fail to result in genetic contribution (Armstrong & Seddon, 2008; Fischer & Lindenmayer, 2000). In addition, evaluating unassisted genetic connectivity among populations can be used to assess whether a population needs a translocation. For example, dispersal was greater than expected among reintroduced elk (*Cervus elaphus*) populations originally thought to be genetically isolated (Hicks et al., 2007). If it is determined that dispersal provides sufficient genetic connectivity to certain populations, wildlife managers could devote resources to augmentation efforts for other populations that are genetically isolated.

After a translocation event, selection, genetic drift, unassisted gene flow, and mutation can influence population evolution on different timescales, making it difficult to identify relative influences on population viability (Frankham et al., 2017). Thus, assessing how translocations affected multiple isolated populations with contrasting translocation histories and situations could help us understand the variation in population evolution (Moraes et al., 2017; White et al., 2018). Genomic techniques can be used to evaluate the success of past translocations and plan future efforts (Bell et al., 2019). Specifically, genomic analyses can help evaluate the genetic effects of previous translocations and assess mean kinship to inform new translocation efforts (Frankham et al., 2017; Jahner et al., 2018). Additionally, comparing long‐term genomic effects of translocations in multiple wild populations would further our understanding of this conservation tool and inform future strategies for genetic management of species with fragmented distributions and genetically isolated populations.

Bighorn sheep (*Ovis canadensis*) in North America continue to face challenges found in many other fragmented wildlife populations, such as low recruitment, poor population growth rates, and widespread disease issues resulting in periodic die‐offs that reduce populations an average of 48% with subsequent prolonged periods of poor lamb survival (Cassirer et al., 2013, 2018; Manlove et al., 2016; Singer et al., 2000). Prior to European expansion across the American west in the late 19th and early 20th centuries, there were an estimated 1.5 to two million bighorn sheep (Seton, 1929). However, market hunting, competition with domestic sheep, and new respiratory diseases introduced by comingling domestic sheep and goats resulted in a decline by 1960 to fewer than 20,000 bighorn sheep in scattered patches across North America (Buechner, 1960; Cassirer et al., 2018; Norris, 1877; Valdez & Krausman, 1999; Whittlesey et al., 2018a, 2018b). Continued concerns regarding resident pathogens in bighorn sheep populations and disease spillover from domestic sheep that may cause epizootic events have resulted in management to keep populations small and isolated (Butler et al., 2018; Cassirer et al., 2016).

Translocation has been the primary management tool used to reintroduce bighorn sheep to previously occupied areas and augment the size and genetic diversity of existing populations. Over 1,460 translocations of 21,500 bighorn sheep have taken place across the indigenous range of this species, and some populations have been studied as examples for genetic rescue (Hogg et al., 2006; Olson et al., 2012; Poirier et al., 2019; Wild Sheep Working Group, 2015). Despite these efforts, restoration of the species continues to be a challenge, as there are still large areas of unoccupied historic range and many small populations with poor demographic performance (Montana Department of Fish, Wildlife, & Parks, 2010).

**Figure 1:**
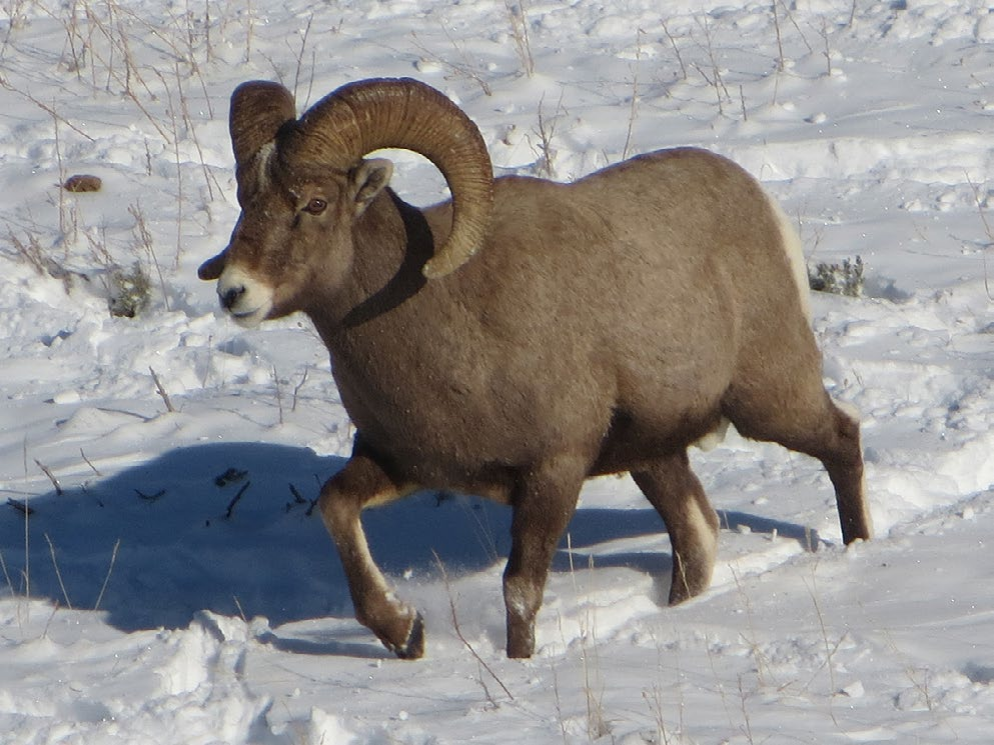
Rocky Mountain bighorn sheep (*O.c. canadensis*) ram from the Taylor Hilgard population in Montana, USA

To inform future translocations and restoration of bighorn sheep, we used a genomic approach to evaluate multiple hypotheses regarding reintroduction, augmentation, and unassisted gene flow of Rocky Mountain bighorn sheep (*Ovis canadensis canadensis*) populations with different origins and translocation histories (Figure 1). The origin of bighorn sheep populations included native (indigenous) herds and reintroduced herds with different founder sizes and number of generations since establishment. Some reintroduced and native herds received augmentations (translocations into an existing herd). Using this diverse set of populations, we evaluated unassisted genetic connectivity within and between herds (i.e., gene flow indicating natural movements of breeding individuals) to determine (a) whether large, spatially structured populations expected to be genetically connected contain barriers to gene flow and (b) whether populations managed and expected to be isolated have genetic contributions due to natural dispersal. Secondly, we evaluated genetic differences between reintroduced herds and their documented founding source to determine (a) whether the population was established via translocation or recolonization and (b) what factors influenced reintroduction success (i.e., lack of signal for recolonization), such as environmental attributes or origin of the source herd. Thirdly, we evaluated genetic differences between reintroduced herds and their founding source to compare alternative hypotheses about which factors influenced the evolution of herds after reintroduction, including founder population size and unassisted/assisted gene flow between herds. Finally, we evaluated relative contributions of past augmentations (i.e., reproductive success of translocated individuals) to determine what factors influenced augmentation success, including the number and sex of translocated animals. We synthesized this information to inform risk and benefit assessments of future augmentations and reintroductions. The results of this study provide insights into long‐term genomic consequences of different translocation approaches used for species restoration and may help inform future genetic management of bighorn sheep.

## 2 MATERIALS AND METHODS

### 2.1 Study populations

**Figure 2:**
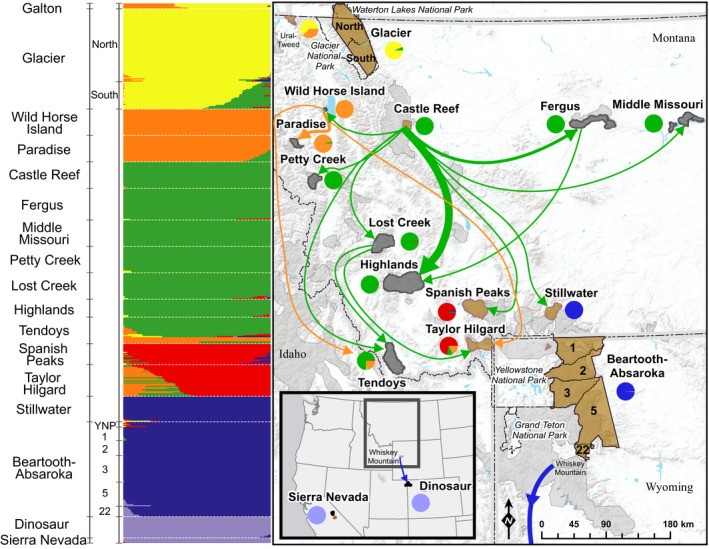
Map of fastStructure (*K* = 6) results for Rocky Mountain (*O.c. canadensis*) and Sierra Nevada (*O.c. sierrae*) bighorn sheep populations genotyped using the HD Ovine array. Approximate distributions of native populations are brown polygons; reintroduced populations are black polygons. A pie chart of herd‐level fastStructure group assignments is next to each population. All known translocation events between Rocky Mountain bighorn sheep populations in this study are shown by arrows. Arrows point generally to the recipient population and do not represent exact release location. Arrow thickness is proportional to number of translocations; arrow color corresponds to the predominant fastStructure group assignment of the source population. Approximate bighorn sheep ranges, including populations not in this study, are shown in gray polygons for Idaho, Wyoming, and Montana (Montana Fish, Wildlife, & Parks, 2008; Thomas, 2019; Wyoming Game & Fish Department, 2012). Hunt unit boundaries for Beartooth‐Absaroka are labeled and truncated to bighorn sheep range. State boundaries are designated by dashed lines outlined in gray; national park boundaries in the study area are designated by dashed lines

We evaluated sixteen Rocky Mountain bighorn sheep populations found in the United States (U.S.) and Canada, including the U.S. states of Montana, Wyoming, Colorado, and Utah, and the Canadian provinces of Alberta and British Columbia (Figure 2). We sought to sample at least 20–25 individuals per population, based on sample size simulations that determined sampling less than this number would introduce an unacceptable level of uncertainty to estimates of genomic kinship between populations (Flesch et al., 2018). We evaluated seven native herds, including one population of 80–90 animals with no augmentations (Galton), three small to moderately sized populations (80–200 animals) with augmentation attempts (Spanish Peaks, Taylor Hilgard, and Stillwater), and three large, continuous populations (380–3,800 animals), including Beartooth‐Absaroka, Castle Reef, and Glacier (Table S1). Castle Reef is a geographic portion of a large, spatially structured population (a collection of subpopulations that occupy distinct geographic areas but are linked by animal movement) and is expected to have connectivity across four administrative units (Figure 2; Montana Department of Fish, Wildlife, & Parks, 2010). Beartooth‐Absaroka and Glacier provided baseline genetic examples of large, spatially structured populations prior to widespread fragmentation of the species’ range without an extensive history of augmentations. Due to large population size and range, we had a greater sample size of 90–95 individuals to represent each of these two herds. The Beartooth‐Absaroka population spans multiple management units along the eastern portion of the Greater Yellowstone Area, including Yellowstone National Park and Wyoming hunt units 1, 2, 3, 5, and 22 (Figure 2). Wyoming units 5 and 22 each received an augmentation from a nearby native population, Whiskey Mountain (Table S2). The Glacier population spans the U.S.–Canada border, including Glacier and Waterton Lakes National Parks. A large lake and adjacent forest forms at least a partial geographic barrier to most bighorn sheep movement within Glacier, so in some analyses we assessed the Glacier population in units north and south of this drainage (Figure 2; Tosa personal communication).

We also evaluated nine reintroduced herds. For eight of those herds, one or all of the initial founding sources were included in this study (Figure 2; Table S2). We considered translocations within 3 years of the first reintroduction event to an unoccupied area to be part of the potential founding source for all populations except for Wild Horse Island. For this population, we considered two translocations that were 8 years apart as founding events, as the first reintroduction event consisted of only two animals. Founder size in reintroduced populations ranged from eight to 53 bighorn sheep. Based on a generation length of 6 years, estimated using the mean age of reproductively active females, there were five to 11 generations since establishment of the reintroduced study populations (Hogg et al., 2006; Johnson et al., 2011). Montana Department of Fish, Wildlife, and Parks (2010) defined five ecological regions that contain bighorn sheep herds within the state of Montana, including prairie/mountain foothills, prairie/breaks, northwest montane, mountain foothills, and southern mountains (Table S1). We initially included the ninth reintroduced population, geographically distant Dinosaur National Monument in northern Colorado and Utah, to serve as a same‐species outgroup, but later learned that some translocations from the previously mentioned Whiskey Mountain herd occurred in this region.

As a true outgroup, we evaluated a different subspecies, Sierra Nevada bighorn sheep (*Ovis canadensis sierrae*; Buchalski et al., 2016; Wehausen et al., 2005). We included five outgroup samples following a similar outgroup approach by Sim et al. (2016). Three Sierra Nevada samples originated from the native Sawmill population, and two samples came from the reintroduced Wheeler population, which was founded by Sawmill individuals (Table S2). We gathered records for all known translocations received by study populations, including translocations that originated from areas not included in the study (Table S2). Where both the source and recipient populations were in this study, approximately zero to eight generations occurred between augmentation and genetic sampling.

### 2.2 Sample collection and DNA extraction

Bighorn sheep were live‐captured for genetic sampling between 2002 and 2018. Each population was sampled over a time span of 1 day to 4 years (less than one generation), except for the Glacier population that was sampled over a period of 9 years (about 1.5 generations). Animal capture and handling protocols were approved by Institutional Care and Use Committees at Montana State University (Permit # 2011‐17, 2014‐32), Montana Department of Fish, Wildlife, and Parks (Permit # 2016‐005), Parks Canada (Permit # WL‐2005‐638), Wyoming Game and Fish Department (Permit # 854), U.S. Geological Survey (Permit # 2004‐01, DINO‐2008‐SCI‐0010), and University of Montana (Permit # 024‐07MHWB‐071807 and 012‐16MMMCWRU‐022916). Genetic samples included FTA Classic gene cards, whole blood samples, biopsy punches from ear cartilage, and muscle or lung tissue from hunter‐harvested or road killed animals. We extracted DNA with Maxwell 16 LEV Blood DNA and Tissue Kits using the Promega Maxwell 16 Magnetic Particle Automated Extractor per kit instructions. For gene cards, we used a modification of the Promega LEV Blood DNA kit. Specifically, we incubated two to three 5 mm gene card punches with proteinase K and lysis buffer in a DNA IQ spin basket (Promega), spun at 3000 XG for 5 min, and loaded the flow‐through into the Maxwell 16 LEV Blood DNA cartridge.

### 2.3 Genomic dataset and quality control

We genotyped bighorn sheep samples using the Illumina High Density (HD) Ovine array, also referred to as a SNP (single nucleotide polymorphism) chip. For genotyping, we used samples with a minimum of 300 ng of DNA, a minimum DNA concentration of 20 ng/µl, and a 260 nm/280 nm ratio of 1.0 to 1.5. The Ovine array is composed of 606,006 SNPs and was originally developed for domestic sheep with a density of one SNP per 4.279 kb, but its development included five bighorn sheep and four Dall's sheep (*Ovis dalli*; Kijas et al., 2014, 2009). Speciation between domestic and bighorn sheep occurred about three million years ago, but the two species can interbreed to produce viable hybrid offspring (Bunch et al., 2006; Young & Manville, 1960). Domestic and bighorn sheep have the same number of chromosomes and are expected to have high genomic synteny (Poissant et al., 2010). An estimated 24,000 SNPs in the HD Ovine array are informative for Rocky Mountain bighorn sheep, and the domestic sheep reference genome enables mapping SNPs to chromosomes (Kohn et al., 2006; Miller et al., 2015). SNP chips can have ascertainment bias, as only a select number of individual samples were evaluated to create the panel and the chip can have an uneven distribution of informative SNPs (Albrechtsen et al., 2010), but Flesch et al. (2018) determined that Rocky Mountain bighorn sheep populations could be differentiated using the HD Ovine array with adequate sample size.

We imported all Illumina genotype data into Golden Helix SNP and Variation Suite v8.6.0 (SNP & Variation Suite, 2016). Using Golden Helix software, we concatenated multiple datasets, mapped the dataset to the domestic sheep reference genome Oar v.4.0, removed samples with a call rate less than 0.85, removed markers on sex chromosomes and with unknown mappings, and exported data into PLINK format for filtering (Purcell et al., 2007; SNP & Variation Suite, 2016). We completed additional filtering and data analysis in the RStudio environment using GNU bash v4.3.48(1) and R v3.4.4 (GNU bash version 4.3.48(1)‐release x^86^_^64^‐pc‐linux‐gnu, 2013; R Core Team, 2017; RStudio Team, 2015). For kinship calculations, we filtered SNPs using a minor allele frequency of less than 0.0001 to remove monomorphic and extremely rare markers, and we removed markers with poor performance by requiring a SNP call rate greater than 0.99 (de Cara et al., 2013; Huisman et al., 2016). The dataset used for the kinship analysis did not require additional filtering of low frequency SNPs prior to analysis based on KING software guidelines (Manichaikul et al., 2010). To infer population structure and ancestry, we further filtered the dataset using a minor allele frequency of 0.01 and a Hardy–Weinberg equilibrium *p*‐value of less than .00001 (Huisman et al., 2016). We used linkage disequilibrium (LD) pruning to remove nonindependent SNPs that informed the presence of nearby variants, using a window size of 100 SNPs, window increment of 25 SNPs, and an LD statistic of *r* ^2^ (Huisman et al., 2016). Code used for filtering and analyses is in Appendix S1.

### 2.4 Population structure, ancestry, and kinship analyses

Because we did not know the degree of genetic similarity among evaluated individuals and populations, we addressed our research objectives using multiple complementary methods that employed differing approaches and assumptions regarding the categorization of individuals into genetic populations. To assess the number of populations (*K* clusters) in our dataset and estimate global ancestry (estimated ancestry proportions from each population for each individual), we used fastStructure software (Pina‐Martins et al., 2017; Pritchard et al., 2000; Raj et al., 2014). This analysis uses clustering to estimate the number of distinct genetic groups and employed a probabilistic analysis to assign individuals to one or more genetic populations without reference to the sampling locations. The STRUCTURE approach assumes that there are a certain number of genetic populations that contain random mating and that these distinct groups have different allele frequencies. Thus, the fastStructure model can provide useful information regarding if animals moved in a translocation successfully bred with the resident population, as global ancestry results can detect hybrids of multiple genetic populations. However, the approach has lower accuracy with uneven sampling and assumes random mating, which is frequently inaccurate for wild populations (Alexander et al., 2009; Frankham et al., 2017; Puechmaille, 2016). In addition, while STRUCTURE models can evaluate admixture, they lack a temporal assessment of fragmentation (Frankham et al., 2017).

Thus, we also completed a nested multidimensional scaling (MDS) analysis using KING v2.1.4 (Manichaikul et al., 2010). In contrast to fastStructure, MDS can identify clusters of populations without assumptions regarding Hardy–Weinberg equilibrium, random mating, or the cause of population structure (i.e., isolation‐by‐distance). This is because MDS does not assign individuals to a genetic population before or after the analysis. Instead, MDS uses an unsupervised approach to reduce the dimensionality of the genomic dataset to an interpretable plot, which allows for visualization of patterns of genomic variation and identification of separation among individual samples to address research questions regarding genetic similarity between individuals and populations. We used a nested approach to understand substructure across multiple levels of organization.

To determine the lineage of reintroduced populations in comparison to their hypothesized founding source, we estimated a bifurcating tree that described differentiation among populations. Because we did not expect most of the evaluated bighorn sheep populations to have dispersal of breeding animals between them, we assigned population identity of individuals based on sampling location. To model differentiation due to genetic drift among these populations defined by geography while still accounting for gene flow, we estimated a maximum likelihood bifurcating tree of populations using Treemix v1.13 (Pickrell & Pritchard, 2012). Treemix uses allele frequencies from each population and Gaussian approximation for genetic drift to estimate a tree that represents each population on a branch (Pickrell & Pritchard, 2012). The amount of genetic drift that occurred among populations is represented by a drift parameter calculated by the Treemix software (Pickrell & Pritchard, 2012). Thus, we could use this analysis to determine the lineage of reintroduced populations, in comparison to their hypothesized founding source. Possible admixture (gene flow) events between branches of the tree were evaluated using a stepwise likelihood approach, where the software searched the tree for the optimal location of each translocation or dispersal event (Pickrell & Pritchard, 2012). To further specifically evaluate gene flow between predefined populations, we conducted a three‐population test (Pickrell & Pritchard, 2012; Reich et al., 2009). A negative value of the *f*_3_ statistic produced by the three‐population test suggests that a specified population is admixed (Pickrell & Pritchard, 2012; Reich et al., 2009). Thus, we used these analyses to specifically identify previously unknown admixture events due to dispersal and confirm genetic contribution of augmentation events.

Finally, we estimated mean kinship between populations to evaluate genetic differences in reintroduced herds from their documented founding source. This information can also inform future augmentation decisions by identifying potential source and recipient populations based on their level of genetic similarity (Ballou & Lacy, 1995; Frankham et al., 2017). Kinship, also called coancestry, represents the probability that two randomly sampled alleles from two individuals are identical by descent (Manichaikul et al., 2010). Mean kinship calculated between populations serves as a measure of population similarity, with higher values interpreted as populations that are more related (Frankham et al., 2017). Thus, genetic differentiation between populations is represented by one minus mean kinship (Frankham et al., 2017). We estimated mean kinship between populations using KING v2.1.4 (Manichaikul et al., 2010). To assess how characteristics of translocations influenced the current kinship between reintroduced populations and their founding source, we evaluated six herd attributes that may affect divergence of reintroduced populations using boxplots. These attributes included founder population size, number of generations since herd establishment, number of augmentations from the founding source, number of source populations, number of augmentations from other sources, and level of connectivity with neighboring herds. See Methods S1 for details regarding described analyses.

## 3 RESULTS

### 3.1 Genomic dataset and quality control

We genotyped 541 bighorn sheep samples using the HD Ovine array, resulting in a dataset composed of 606,006 SNPs. Using a sample call rate of 0.85, we filtered 30 samples from the dataset and subsequently evaluated 511 samples from 17 different populations. We met our sample size goal of at least 20–25 bighorn sheep for fourteen Rocky Mountain bighorn sheep populations, excluding Highlands and Galton (Table 1). We included Highlands and Galton populations in the MDS and fastStructure analyses, where overall sample size was less likely to affect the results, but excluded these two low sample size populations from the Treemix analysis and interpreted their mean kinship results with caution (Highlands *n* = 17; Galton *n* = 5). After filtering, 33,289 SNPs were used for kinship estimates; 6,155 SNPs were used for the remaining analyses (Results S1). The dataset used for the kinship analysis did not require additional filtering of low frequency SNPs prior to analysis based on KING software guidelines (Manichaikul et al., 2010).

**Table 1:**
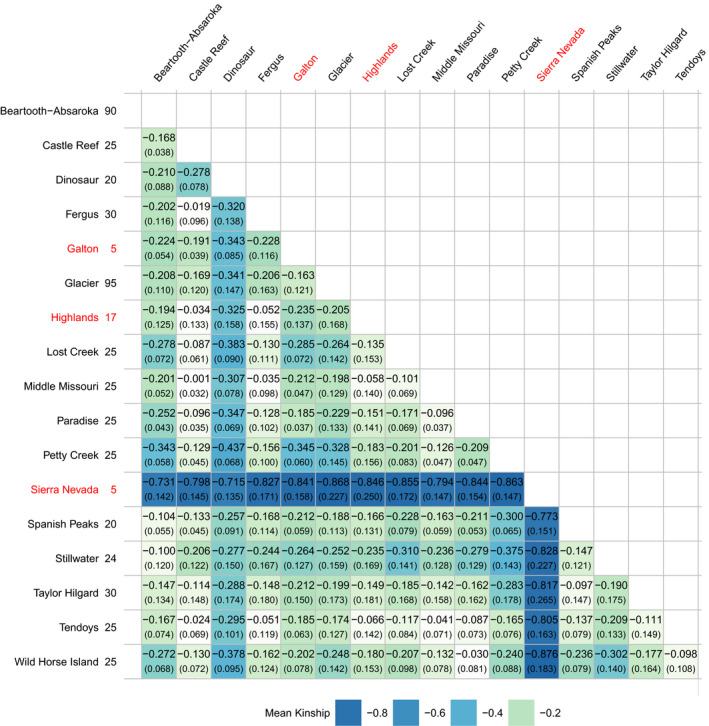
Mean kinship between Rocky Mountain and Sierra Nevada bighorn sheep populations evaluated using the HD Ovine array. Standard deviation from the mean is in parentheses. Smaller values indicate lower mean kinship. Sample size for each population is next to each population name on the y‐axis; populations with fewer than 20 genotypes are labeled in red

### 3.2 Population structure

To evaluate population structure, we estimated the number of genetic populations and ancestry proportions for each individual and population using a fastStructure analysis. Our fastStructure analysis of seventeen bighorn sheep populations suggested there were six genetic clusters (*K*; Results S1). Three clusters consisted of pairs of native populations that were geographically proximate, including Glacier with Galton, Spanish Peaks with Taylor Hilgard, and Stillwater with Beartooth‐Absaroka. Two other genetic groups encompassed source and receiving populations of reintroductions. Wild Horse Island and Paradise formed a distinct cluster because Wild Horse Island was the sole source for the Paradise reintroduction and later augmentation. The Castle Reef cluster encompassed the greatest number of populations, as Castle Reef bighorn sheep or their descendants founded the other populations in the genetic group, including Fergus, Middle Missouri, Petty Creek, Lost Creek, Highlands, and Tendoys. The sixth cluster included geographically distant Dinosaur and outgroup Sierra Nevada. In the *K* = 7 analysis, Petty Creek formed its own cluster, and in the *K* = 9 analysis, Lost Creek formed its own cluster, which may be due to past augmentation from sources not included in this study (Figure S1; Table S2).

We detected past augmentations between genetic groups, where the recipient population contained partial ancestry from the cluster of the source population and the populations were geographically distant, such that movement of breeding animals between the areas was unlikely. We detected the following translocation events, indicating that translocated individuals survived and reproduced successfully at the release site: Wild Horse Island to Taylor Hilgard, Lost Creek to Taylor Hilgard, Wild Horse Island to Tendoys, and Castle Reef to Tendoys (Figure 2; Figure S1). The success of an augmentation from Castle Reef to Spanish Peaks was unclear, as we detected genetic ancestry from the cluster including Castle Reef in fewer than five individuals in Spanish Peaks (Figure S1). We also detected augmentations from populations that were not directly sampled but were also augmentation sources to herds in our study. The Whiskey Mountain population (Figure 2; Wyoming hunt unit 10) provided two augmentations that likely made a genetic contribution to Wyoming hunt units 5 and 22 in the Beartooth‐Absaroka (Love Stowell et al., 2020; Wild Sheep Working Group, 2015). Managers moved individuals from Whiskey Mountain to augment the reintroduced Dinosaur population three times, which was detected by a shared cluster between Dinosaur and augmented Beartooth‐Absaroka hunt units 5 and 22. Finally, our results suggested historical gene flow between the Glacier population and geographically proximate Castle Reef population, as Castle Reef bighorn sheep shared genetic ancestry from the same cluster with the southern unit of the Glacier population.

### 3.3 Genetic distinctiveness

**Figure 3:**
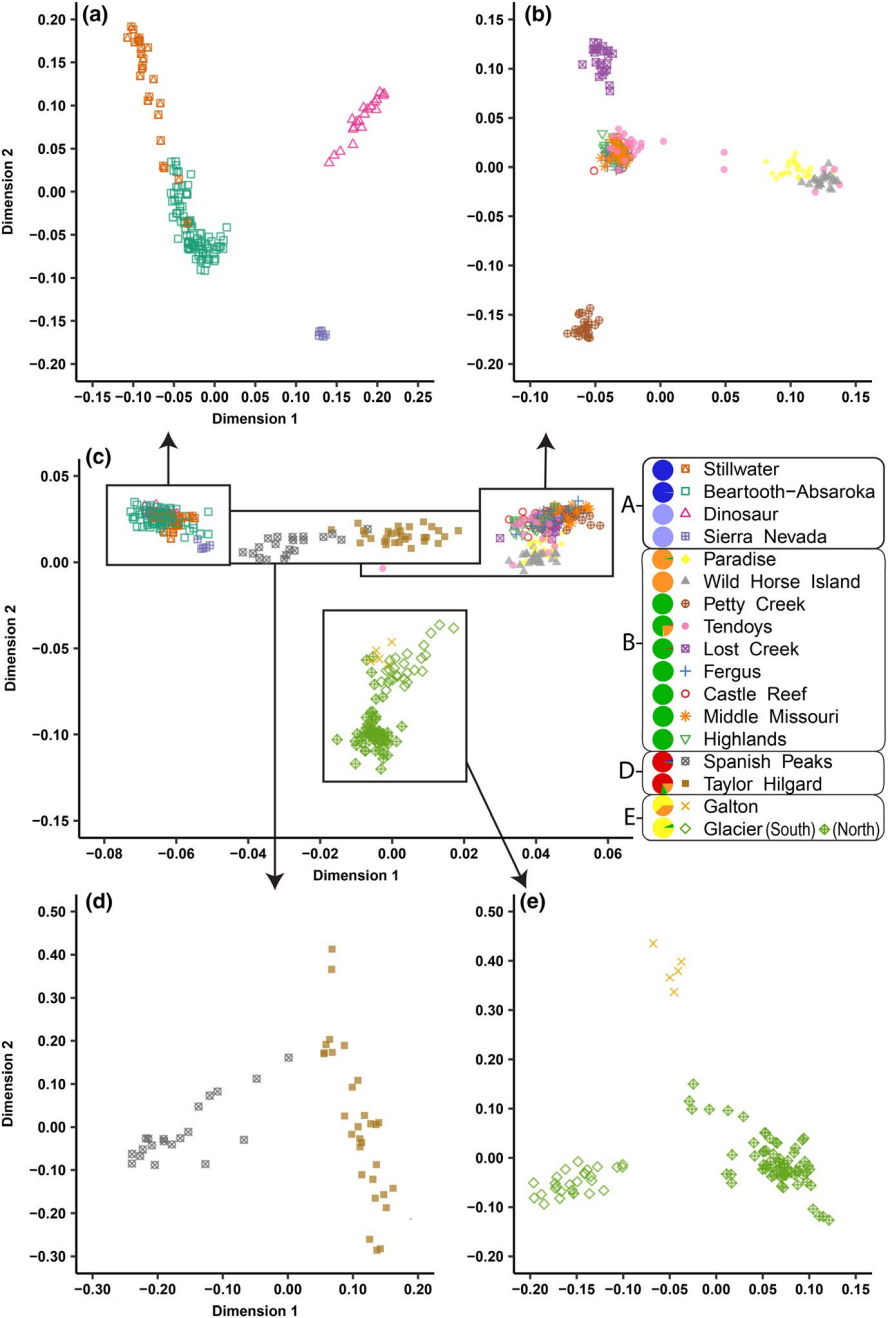
Multidimensional scaling (MDS) results for individual HD Ovine array genotypes from seventeen bighorn sheep populations, including an analysis of all herds (c) and subset analyses (a, b, d, and e) based on clusters of populations in panel c. The legend defines symbols for each population and pie charts of herd‐level fastStructure group assignments (*K* = 6)

We used the MDS analysis to further evaluate clusters of populations in a hierarchical fashion without assumptions required by the fastStructure software. We identified four clusters of populations in the MDS analysis and evaluated the four MDS clusters separately (Figure 3). The cluster shown in Figure 3a encompasses all populations found outside of Montana, including the Sierra Nevada, Dinosaur, and the Beartooth‐Absaroka, as well as Stillwater, which is located in Montana but near the Montana‐Wyoming border. Most Stillwater individuals separated from the Beartooth‐Absaroka, although the two populations were assigned into a single fastStructure cluster. However, four Stillwater individuals did not separate from Beartooth‐Absaroka genotypes. Outgroup Sierra Nevada and geographically distant Dinosaur each separated from the other populations.

Figure 3b, which generally depicts the extensive history of translocations from Castle Reef, included nine out of seventeen populations, similar to the fastStructure results. Wild Horse Island, Lost Creek, and Petty Creek populations were all founded by Castle Reef animals but separated more from their founding source than the Fergus, Middle Missouri, and Highlands populations, indicating different influences on the evolution of these populations. The Paradise population separated somewhat from its founding and only augmentation source, Wild Horse Island. Results for individuals from the Tendoys reflected the herd's translocation history from five different sources. Most individuals in the Tendoys clustered with the Castle Reef population, which provided an augmentation, rather than the Lost Creek population, which founded the Tendoys population. Four Tendoys individuals clustered with Wild Horse Island, the source of one augmentation. Three Tendoys individuals plotted partway between Castle Reef/Lost Creek and Wild Horse Island clusters, likely representing hybrids from these two lineages. One Tendoy individual had an outlier genotype in the overall analysis (Figure 3c), which may represent an individual descended from neighboring populations in Idaho or two augmentations from areas not in the study (Table S2).

Figure 3d includes the Taylor Hilgard and Spanish Peaks populations, which separated in the subset MDS analysis, despite being grouped into the same fastStructure cluster. The separation of the two populations into two separate genetic clusters in the MDS analysis suggested a lack of current genetic connectivity. When compared to all other populations, Spanish Peaks plotted closer to the nearby native populations of Stillwater and Beartooth‐Absaroka, whereas Taylor Hilgard plotted closer to its augmentation sources, Lost Creek and Wild Horse Island (Figure 3c). Figure 3e includes Glacier and Galton, where Glacier genotypes generally separated within the population based on location, north and south of a large lake drainage, and Galton was distinct from Glacier.

### 3.4 Population tree and genetic contributions from augmentations

**Figure 4:**
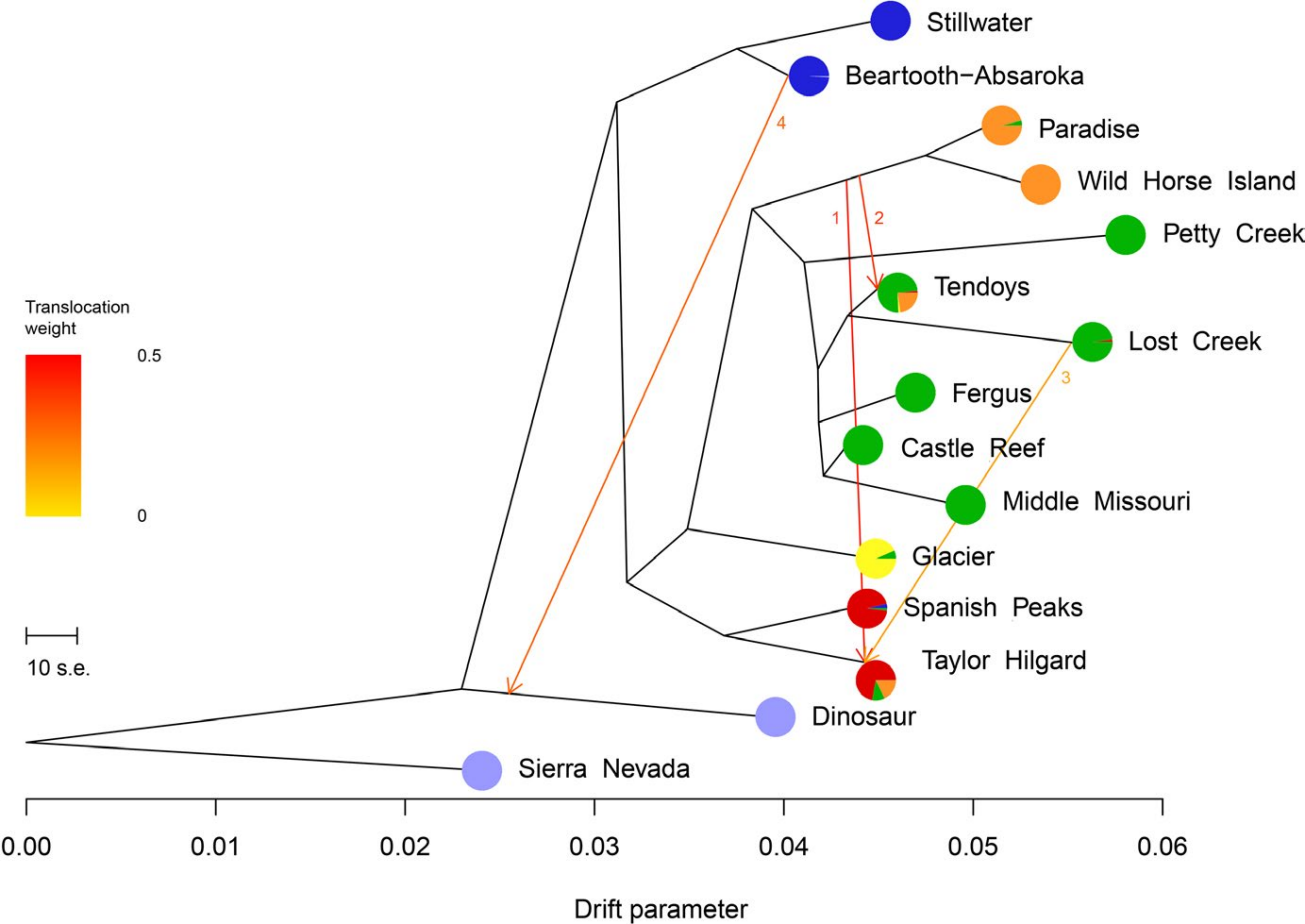
Treemix population tree with four detected translocations (orange lines) plotted for fourteen Rocky Mountain bighorn sheep populations with 20 or more genotypes. Sierra Nevada was defined as the outgroup. Horizontal axis scale bar defines ten times the mean standard error of the sample covariance matrix; horizontal branch length is proportional to genetic drift amount. Pie charts of herd‐level fastStructure group assignments (*K* = 6) are shown for each population

We used Treemix to evaluate the lineages of populations defined by geography using a bifurcating tree that accounted for gene flow. The population tree generated by Treemix was consistent with fastStructure, as the nodes on the tree generally grouped together populations found in the same fastStructure clusters (Figure 4). Sierra Nevada was defined as the outgroup, and detailed information about Treemix model selection can be found in Results S1. Geographically distant Dinosaur was the least related to all other evaluated Rocky Mountain bighorn sheep populations. Stillwater and Beartooth‐Absaroka were grouped together, followed by Spanish Peaks and the Taylor Hilgard. Glacier formed its own branch, as Galton was not evaluated due to low sample size. The remaining populations grouped together were influenced by a history of translocations from Castle Reef and its descendant populations. The population founded by Castle Reef that showed the highest divergence was Wild Horse Island, and the founding of Paradise by Wild Horse Island was accurately represented in the population tree. Reintroduction of the Tendoys herd by founders from Lost Creek was depicted in the population tree, despite the possibility for genetic connectivity with neighboring herds in Idaho and later augmentations directly from Castle Reef. Petty Creek and Lost Creek showed greater drift parameters from Castle Reef than Fergus and Middle Missouri, consistent with MDS and fastStructure results, although this may be because these populations received augmentations from other herds not included in this study, rather than genetic drift.

The Treemix model identified four augmentation events between specific populations (Figure 4, orange lines; Table 2). All four identified gene flow events represented known augmentations where shared ancestry between populations was identified by fastStructure (Tables 2 and S2). The direction of the plotted augmentation event was the least stable feature of the Treemix analysis, and we reversed the direction of augmentation events identified 3rd and 4th, as the direction was known and unassisted gene flow was unlikely due to geographic separation of the identified areas (Tables 2 and S2). Translocation weight estimated the proportion of alleles contributed by the source population, assuming admixture occurred in one generation (Pickrell & Pritchard, 2012). The three‐population test, which tested for admixture between populations defined by geography, further supported genetic contributions of Wild Horse Island, Castle Reef, and Lost Creek to the Tendoys from translocations (Table S3). In addition, the three‐population test suggested unassisted gene flow (natural movement of breeding animals) between Stillwater and the Beartooth‐Absaroka, consistent with Figure 3a.

**Table 2:** Translocation events identified by Treemix based on the population tree (Figure 4)

Translocation order	Source	Recipient	Translocation weight
1	Wild Horse Island	Taylor Hilgard	0.45
2	Wild Horse Island	Tendoys	0.39
3	Lost Creek	Taylor Hilgard	0.15
4	Beartooth‐Absaroka	Dinosaur	0.29

Note

Translocation weight can range from 0 to 0.5 and estimated the proportion of alleles contributed by the source population.

### 3.5 Comparing translocation history and genomic analyses

To identify which reintroduction and augmentation efforts made a genetic contribution to the recipient population, we synthesized genomic evidence from fastStructure, MDS, and Treemix for 24 different translocation events where both populations were included in the study (Table 3). For all eight reintroduced herds with founding source data, genetic contribution of the suspected founding group(s) to the contemporary population was suggested by at least two analyses. Fifteen out of 24 translocations were augmentations, including 11 unique pairs of source and recipient populations. Four out of 11 augmentation pairs could not be assessed for genetic contribution, as the source herd was the same as or genetically similar to the founding source. Of the remaining seven source and recipient augmentation pairs, we detected five augmentation pairs in multiple analyses, including Wild Horse Island to Taylor Hilgard, Lost Creek to Taylor Hilgard, Wild Horse Island to Tendoys, Castle Reef to Tendoys, and Beartooth‐Absaroka (due to augmentation from Whiskey Mountain) to Dinosaur. Augmentations to Spanish Peaks and Stillwater from Castle Reef were not detected in multiple analyses, suggesting that after these two augmentation events, the translocated individuals did not survive or reproduce.

**Table 3:** Evidence for all known translocations between herds in this study detected using MDS (multidimensional scaling), fastStructure, and Treemix analyses

Herd receiving animals	Herd providing animals	Year	Number animals	Translocation details	MDS	fastStructure	Treemix
Dinosaur	Beartooth‐Absaroka	1983	19	Augmentation of reintroduced herd	Possible	Detected	Detected
Dinosaur	Beartooth‐Absaroka	1984	17	Augmentation of reintroduced herd			
Dinosaur	Beartooth‐Absaroka	1989	21	Augmentation of reintroduced herd			
Fergus	Castle Reef	1961	12	Founder	Possible	Detected	Possible
Fergus	Castle Reef	1980	28	Augmentation from founder source			
Highlands	Castle Reef	1967	22	Founder 1	Possible	Possible	Not evaluated
Highlands	Castle Reef	1969	31	Founder 2			
Highlands	Castle Reef	2000	15	Augmentation from founder source			
Highlands	Castle Reef	2001	17	Augmentation from founder source			
Highlands	Castle Reef	2008	65	Augmentation from founder source			
Highlands	Fergus	2014	9	Augmentation of reintroduced herd	Possible	Possible	Not evaluated
Lost Creek	Castle Reef	1967	25	Founder	Possible	Detected	Possible
Middle Missouri	Castle Reef	1980	28	Founder	Possible	Detected	Possible
Paradise	Wild Horse Island	1979	14	Founder	Possible	Detected	Possible
Paradise	Wild Horse Island	2011	22	Augmentation from founder source			
Petty Creek	Castle Reef	1968	16	Founder	Possible	Detected	Possible
Spanish Peaks	Castle Reef	1974	2	Augmentation of native herd	Not detected	Possible	Not detected
Stillwater	Castle Reef	1970	2*	Augmentation of native herd	Not detected	Not detected	Not detected
Taylor Hilgard	Lost Creek	1989	18	Augmentation of native herd	Not detected	Detected	Detected
Taylor Hilgard	Wild Horse Island	1993	26	Augmentation of native herd	Not detected	Detected	Detected
Tendoys	Lost Creek	1984	39	Founder	Possible	Detected	Possible
Tendoys	Castle Reef	2002	30	Augmentation of reintroduced herd	Possible	Detected	Possible
Tendoys	Wild Horse Island	2012	49	Augmentation of reintroduced herd	Possible	Detected	Detected
Wild Horse Island	Castle Reef	1947	6	Founder	Possible	Not detected	Possible

Note

An asterisk after the total number of bighorn sheep indicates that biologists suspected the translocation failed and did not contribute to the receiving population. For the MDS analysis (Figure 3), a translocation detection was designated as “possible” if both herds were found in the same group that was evaluated in a subset MDS analysis and “not detected” if herds were not evaluated together in a subset MDS analysis. For fastStructure (Figure 2), a translocation was considered “detected” if a common cluster was found for more than five animals in either herd, “possible” if a common cluster was found for less than five animals in either herd or there was an alternative source herd within the same cluster as the herd providing animals, and “not detected” if a common cluster was not found in both herds. For Treemix (Figure 4), a translocation was considered “detected” if identified as a migration event by the software, “possible” if herds were plotted on nearby branches in the tree, and “not detected” if herds were not plotted on nearby branches in the tree.

### 3.6 Genetic similarity

We estimated mean kinship between bighorn sheep populations to evaluate genetic similarity and inform future augmentation efforts based on mean kinship (Table 1). Mean kinship values ranged from −0.868 (most unrelated) to −0.001 (most related). All mean kinship values between herds were negative, indicating that allelic correlations were less than expected under an assumption of Hardy–Weinberg equilibrium (Frankham et al., 2017). A lack of Hardy–Weinberg equilibrium between herds was consistent with the geographic isolation of most populations and a limited number of recent translocations (Figure 2; Table S2). For example, a negative mean kinship value for the level of similarity between Taylor Hilgard and Spanish Peaks supported the idea that there is a lack of current genetic connectivity between the two populations, also suggested by the MDS analysis. The Sierra Nevada outgroup and geographically distant Dinosaur population had the lowest mean kinship with all other populations. The highest mean kinship value was found between the Middle Missouri and Castle Reef populations (−0.001, standard deviation of 0.032), likely because the Middle Missouri herd was founded by one translocation of 28 Castle Reef bighorn sheep in 1980 and had no other augmentations.

We estimated mean kinship between reintroduced herds and their founding source to evaluate six attributes that could affect population evolution since reintroduction (Figure S4). These attributes included founder population size, number of generations since herd establishment, number of augmentations from the founding source, number of source populations, number of augmentations from other sources, and level of connectivity with neighboring herds. As the number of augmentations from other areas and the number of source populations increased, mean kinship with the founding source generally decreased (Figure S4d,e). All six examined herd attributes likely influenced evolution of reintroduced herds to differing extents, which complicated our interpretation of which attributes were dominant. However, application of this approach with a greater sample size of populations could serve as a method to further evaluate which population attributes influenced reintroduced population evolution and genetic divergence from the founding source. See Results S1 for details.

## 4 DISCUSSION

We examined the genomic consequences of bighorn sheep restoration to enhance understanding of evolutionary processes such as gene flow from genetic connectivity or genetic drift from isolation that affect population genetics and to inform genetic management of fragmented populations through future translocation strategies. First, our genetic structure results indicated genetic connectivity due to natural dispersal of individuals within large, spatially structured populations, but not between most populations. Second, genetic similarity between reintroduced populations and their documented founding source indicated that translocation, rather than recolonization, was the source of all populations initially expected to be reintroduced. Third, genetic similarity between reintroduced populations and their founding sources was mainly influenced by later augmentations, rather than founding population size. Fourth, we detected genetic contributions from augmentations consisting of 18–57 animals including males and females, but not from augmentations of two males. This information can guide selection of source populations and translocation strategies to aid in restoration of bighorn sheep.

### 4.1 Natural genetic connectivity detected only in large populations

Genetic structure and connectivity across native populations prior to fragmentation due to human activities can serve as a baseline goal for bighorn sheep restoration in other areas. Glacier and Beartooth‐Absaroka served as examples of large, continuous native populations, and our results suggested genetic connectivity within these populations and that gene flow within these herds was influenced by geographic distance and at least one natural barrier. Genetic differences between the north and south portions of Glacier suggested partial fragmentation due to a large, central lake that may serve as a barrier to extensive gene flow (Figures 2 and 3). In addition, our results suggested isolation‐by‐distance within Stillwater and Beartooth‐Absaroka, as results for Stillwater, Yellowstone, northern hunt units 1–3, and southern hunt units 5 and 22 suggested small genetic differences within a larger population (Figures 2 and 3). Love Stowell et al. (2020) also detected a north‐south pattern of isolation‐by‐distance in the Beartooth‐Absaroka population. Between populations, distances over 100 km and noncontiguous mountain ranges generally reduced detection of past and present unassisted gene flow. For example, native Galton and Castle Reef were genetically different and about 170 km apart in linear distance (Figure 2). Populations outside of Montana and Wyoming, including Sierra Nevada and Dinosaur, were distinct from one another and all other populations (Figure 3). In contrast, our results for restored herds did not suggest isolation‐by‐distance, but instead a strong genetic influence of translocations. This is consistent with patterns observed in restored white‐tailed deer (*Odocoileus virginianus*) populations, where little genetic differentiation existed due to translocations to repopulate previously occupied areas (Budd et al., 2018).

Our results identified native populations that had historical gene flow but likely did not have genetic connectivity at the time of sampling. To identify populations that were recently fragmented, we evaluated MDS results, as Frankham et al. (2017) recommended identifying clusters of populations using MDS to evaluate recent gene flow. In addition, we evaluated the three‐population test results, because a three‐population test can specifically evaluate gene flow between predefined populations (Pickrell & Pritchard, 2012; Reich et al., 2009). Our MDS and three‐population test results suggested that most populations were genetically isolated from one another, except for Stillwater and Beartooth‐Absaroka. This is likely because Stillwater and Beartooth‐Absaroka are in geographic proximity and part of a continuous, spatially structured population. Although female bighorn sheep often associate in genetically related groups (Boyce et al., 1999; Rubin et al., 1998), rams often disperse during breeding season to genetically connect ewe groups (Geist, 1971). Our genotyping approach accounted for male genetic contributions. In addition, for most populations we sampled animals across a broad distribution via helicopter search and capture. Thus, we expect that our results represent genetic isolation among examined herds, rather than genetic subgroups within a larger population. However, examined herds could have gene flow with nearby populations not evaluated in this study, and additional genetic sampling of neighboring herds may be useful to more thoroughly evaluate genetic isolation in specific populations.

Gaps between formerly connected populations are areas where managers could prioritize reestablishing connectivity. Filling gaps in distribution can be an important part of species restoration, in addition to establishing new populations (Stewart et al., 2017; Watson & Watson, 2015). Broadly distributed and spatially structured populations can also help prevent extirpation of an entire population after an epizootic by localizing outbreaks in smaller groups and lowering probability of disease spread (Altizer et al., 2003; Lopez et al., 2005). We detected two pairs of native herds that had past genetic connectivity but were likely not connected at the time of sampling, including Spanish Peaks/Taylor Hilgard and Castle Reef/south Glacier. These populations were similar in fastStructure and Treemix analyses, but the three‐population test and MDS suggested a lack of contemporary gene flow. The linear distances between recently fragmented populations were comparable to those for herds with recent gene flow. Stillwater and Beartooth‐Absaroka are about 25 km apart in linear distance, whereas about 35 km separate south Glacier and the nearest bighorn sheep range connected to Castle Reef and 28 km separate Spanish Peaks and Taylor Hilgard. However, the lack of signal for genetic connectivity between the Glacier and Castle Reef populations may be due to a lack of sampling of animals in the bighorn sheep range closest to Glacier which is likely connected to Castle Reef.

### 4.2 Translocation, not recolonization, started all populations thought to be reintroduced

To evaluate evolution of newly founded populations and inform reintroduction planning, we compared herds thought to be reintroduced with their suspected founding source. Other studies suggested that bighorn sheep reintroduction efforts may be more successful when founders are sourced from either matching environmental conditions, due to greater recruitment when ecotypes are matched, or native populations, due to typically higher levels of genetic diversity (Bleich et al., 2018; Fitzsimmons et al., 1997; Singer et al., 2000). We evaluated eight populations that originated from reintroductions where a founding source was in our study. Castle Reef was the source of six out of eight reintroduced herds, and the remaining two were started by reintroduced populations initially founded by Castle Reef animals that were translocated (i.e., moved by managers to an formerly occupied area), rather than recolonization (i.e., founded by natural dispersal from nearby populations). This result was suggested by each of our three main analyses. In the fastStructure analysis, six of the reintroduced herds shared the same genetic cluster as Castle Reef (Figure 2). In the MDS analysis, eight reintroduced herds clustered with Castle Reef (Figure 3c). In the population tree, the same reintroduced herds that had sufficient sample size to be included in the analysis (seven out of eight populations) shared a common node with Castle Reef that excluded all other evaluated populations (Figure 4). If the populations were established by recolonization, we would not have expected to see consistent genetic similarity with their suspected founding source across multiple analyses. The overall success of reintroductions from Castle Reef suggested that sourcing from the native population may have been more important than matching environmental conditions. Sourced from mountain foothills, Castle Reef animals successfully established populations not only in environments similar to the source location (Lost Creek and Highlands), but also in semiarid prairie/river breaks environments (Fergus and Middle Missouri) and an island with weather influenced by the Pacific Ocean maritime effect (Wild Horse Island; Montana Department of Fish, Wildlife, & Parks, 2010). Translocated bighorn sheep establishing populations in areas with different environmental conditions than their source have been documented in other studies (Malaney et al., 2015; Rominger et al., 2004; Wiedmann & Sargeant, 2014). For example, translocated bighorn sheep can adjust the timing of parturition to match the environmental characteristics of a new area (Whiting et al., 2011, 2012). Local adaptation in bighorn sheep herds is still possible (Wiedmann & Sargeant, 2014), given that native populations found in different ecological regions were genetically differentiated, but genetic distinctiveness could also be explained by genetic drift (Figure 2). Future research to evaluate the possibility for local adaptation would involve assessing patterns of correlation between individual genotypes and environmental characteristics across space, for which there are many analytical approaches (Balkenhol et al., 2017; Rellstab et al., 2015; Selmoni et al., 2019). If ecologically matched native herds have low genetic diversity or are not available for a reintroduction, multiple sources could be used, as maximizing genetic diversity might increase adaptive potential in a new environment (Broadhurst et al., 2008; Olson et al., 2013; White et al., 2018).

### 4.3 Genetic divergence of reintroduced populations from their founding source was mainly influenced by augmentations

After selecting source population(s) for reintroduction, geneticists recommend using a large number of founders to replicate the genetic profile of the founding source, but recommendations can range from 20 to 50 animals and depend on the species (Jamieson & Lacy, 2012; Taylor & Jamieson, 2008; Weeks et al., 2011). It is useful to evaluate how much reintroduced populations genetically diverged from their founding source, to assess whether the released number of founders successfully represented the source's genetic profile, and to determine whether genetic drift occurred, which is the main process by which small populations lose genetic variation, as in reintroduced populations of Alpine ibex (*Capra ibex ibex*; Biebach & Keller, 2009; Lacy, 1987; Templeton, 2006). Thus, greater genetic divergence from the source population may indicate a loss of genetic variation in the reintroduced herd due to chance. We expected that reintroduced populations established with a greater number of founders would have greater mean kinship with the founding source, as a large number of animals would be more genetically representative of the source and experience a lower rate of genetic drift. When we compared founder sizes ranging from six to 53 bighorn sheep to kinship with the founding source, we did not find a clear relationship (Figure S4a). For some populations, a limited number of generations have passed since founding; thus, genetic drift may not yet be detectable (Figure S4b). Kinship with the founding source may also have been driven by other genetic influences in many of the reintroduced herds, as six out of eight reintroduced populations received translocations from at least two sources (Figure S4d). Three reintroduced populations that had the greatest divergence from their founding source (Wild Horse Island, Lost Creek, and Petty Creek) each were founded by 25 or fewer animals from the same source, received an augmentation from at least one other source, and had 8–11 generations for genetic drift or selection. Thus, a founder effect, genetic drift or selection over time, and genetic input from a different source likely influenced population genetics of these reintroduced populations. In contrast, the population with the highest mean kinship with its founding source (Middle Missouri) had one reintroduction event of 28 animals, six generations of isolation, and no augmentations. While multiple influences on the evolution of reintroduced populations complicated identifying which affected divergence the most, we observed an inverse relationship between the number of source populations and augmentations from other sources and mean kinship of reintroduced populations with their founding source (Figure S4d,e), suggesting that this influence was important in our study. Future genomic studies could target reintroduced populations with different founder sizes and no augmentations to gain clearer insights to inform the number of bighorn sheep recommended for reintroductions.

### 4.4 Augmentations of two males were less successful than larger, mixed sex groups

Augmentations provide an opportunity to assess the impact of specific gene flow events on fragmented population evolution and inform animal selection for future efforts. For translocation to be used for augmenting genetic diversity of small populations, managers need to (a) identify which populations need augmentation based on demographic attributes and levels of population fragmentation, genetic diversity, and inbreeding, (b) identify source populations based on mean kinship, other direct measures of diversity, and other management concerns like disease history, (c) determine how many and which individuals should be moved, and (d) monitor recipient populations for realized genetic contribution, connectivity with nearby populations, genetic diversity, and demographic response, to determine if additional augmentations are needed (Adams et al., 2011; Frankham et al., 2017; Harrisson et al., 2016; Johnson et al., 2010). Future research could evaluate the relationship between levels of inbreeding within herds and population performance to aid in determining which populations may require augmentation.

Identifying which animals will contribute genetically to a recipient population includes considerations such as adaptation, number, sex, age, and disease status. To synthesize results across the fastStructure, MDS, and Treemix analyses, we defined a translocation event as detected only if we found evidence for its genetic contribution in the intended recipient population in at least two out of three analyses (Table 3). As in reintroductions, we observed successful augmentations (defined as genetically detected) across different ecological regions defined by Montana Fish, Wildlife, and Parks and extensive latitudes from the Whiskey Mountain population in Wyoming to the Dinosaur population in Colorado and Utah, suggesting that matching environmental attributes is not always necessary. Because our definition of augmentation success was based on detected genetic contribution and not individual fitness, additional assessment evaluating hybrid fitness would be valuable (Olson et al., 2012).

We found that five detected translocations consisted of 18 to 57 animals, with the following age and sex compositions: three adult males and 27 adult females; five adult males, 40 adult females, and four juveniles of unknown sex; eight adult males, 23 adult females, four male juveniles, five female juveniles, and 17 animals of unknown age and sex; and two translocations with unknown age and sex composition. In contrast, two undetected augmentations each consisted of two males. During breeding season, adult male bighorn sheep can wander long distances between mountain ranges (Geist, 1971) and potentially depart the augmentation destination, which may result in no genetic contribution to the intended recipient population. In addition, because bighorn sheep are a polygynous species, a small number of dominant rams may competitively exclude translocated males due to female mate preference for residents or poor condition after transport/release, suggesting that translocating a greater proportion of females may be more effective for augmentation (Mulder et al., 2017; Sigg et al., 2005). After an augmentation of females, translocated ewe groups may socially segregate from the resident population following release, which was observed in Taylor Hilgard after augmentation from Lost Creek in 1989 (Robinson et al., 2019; Roy & Irby, 1994). However, observed mixing of rams and ewes with different origins during breeding season or social mixing in later years resulted in hybrids descended from native and translocated animals after 24 years or about four generations (Figures 4 and S1; Roy & Irby, 1994). Future research could more specifically evaluate if translocated bighorn sheep males or females are more likely to reproduce successfully.

Augmentations are often promoted for genetic rescue (Hogg et al., 2006; Tallmon et al., 2004; Whiteley et al., 2015). To increase effectiveness of a genetic rescue, source and recipient populations should have been previously connected but recently isolated to allow differentiation over multiple generations in the past 500 generations (Allendorf & Luikart, 2009; Falconer et al., 1996; Frankham et al., 2017). Many types of information should be evaluated to determine optimal sources for augmentation. Our results can provide guidance on selecting sources for genetic rescue augmentations by combining information on genetic differentiation and mean kinship. One possible approach, if managers want to maintain populations that are currently differentiated, would be to identify possible sources for future augmentations within clusters of populations in MDS or fastStructure analyses (Figures 2 and 3). Within identified clusters, managers can select a source population that has low mean kinship with the intended recipient herd (Table 1). Minimizing mean kinship between source and recipient populations within identified clusters would be an approach to retain genetic diversity and minimize inbreeding at the population level while still considering the possibility for local adaptation (Ballou & Lacy, 1995; Frankham et al., 2017). For example, augmentations could be implemented within the cluster associated with Castle Reef (Figure 2), and mean kinship minimized between source and recipient by consulting a mean kinship table associated with that cluster (Table S4).

## 5 CONCLUSIONS AND MANAGEMENT IMPLICATIONS

Our results provide insight regarding the population genomics of native and reintroduced herds, genetic contributions of past translocation efforts, and strategies for future bighorn sheep restoration efforts. This research serves as an example of how genomic analyses can provide information regarding the genetic outcomes of previous management approaches and inform future decisions. Building on other studies that drew conclusions from only a few populations or limited genetic markers, our study design maximized insight from an observational study by employing standardized sampling of fourteen bighorn sheep herds with differing management histories distributed across the northern Rocky Mountain region, a standardized set of SNP markers, and a suite of contemporary analytical tools. Successful genetic contribution of most reintroduction and augmentation efforts evaluated in this study in the context of the continued struggle of Rocky Mountain bighorn sheep conservation suggests there are multiple interacting influences on restoration success. Not only is there uncertainty regarding how genetic attributes affect population performance, but the specific drivers behind population trend and demography are also frequently unclear. Thus, genetic management should generally be integrated into conservation planning with other management considerations (IUCN/SSC, 2013; Ralls et al., 2017). Assessment of multiple population attributes during restoration efforts, such as genetics, migratory patterns, mortality causes, and disease, can enable an adaptive management framework and improve the longevity of managed populations (IUCN/SSC, 2013). Genomic data alone cannot dictate decisions concerning population and genetic management, but rather can be integrated with management judgements as to what population genetics are valuable to conserve and what should be built for the future. Our results demonstrate that genomic analyses are a tool for evaluating the genetic effects of translocations and planning future genetic management of small, fragmented populations.

## CONFLICT OF INTEREST

None declared.

## AUTHOR CONTRIBUTION

**Elizabeth P. Flesch:** Conceptualization (equal); Data curation (equal); Formal analysis (lead); Funding acquisition (equal); Investigation (equal); Methodology (lead); Visualization (lead); Writing‐original draft (lead); Writing‐review & editing (lead). **Tabitha A. Graves:** Data curation (equal); Formal analysis (equal); Funding acquisition (equal); Investigation (equal); Methodology (equal); Resources (equal); Supervision (equal); Writing‐review & editing (equal). **Jennifer Thomson:** Conceptualization (equal); Data curation (equal); Formal analysis (equal); Funding acquisition (equal); Investigation (equal); Methodology (equal); Project administration (equal); Resources (equal); Supervision (equal); Writing‐review & editing (equal). **Kelly M. Proffitt:** Data curation (equal); Funding acquisition (supporting); Investigation (equal); Writing‐review & editing (equal). **PJ White:** Funding acquisition (equal); Project administration (equal); Resources (equal); Writing‐review & editing (equal). **Thomas R. Stephenson:** Data curation (equal); Funding acquisition (equal); Resources (equal); Writing‐review & editing (equal). **Robert A Garrott:** Conceptualization (equal); Data curation (equal); Formal analysis (supporting); Funding acquisition (equal); Investigation (equal); Methodology (equal); Project administration (lead); Resources (equal); Supervision (equal); Writing‐original draft (supporting); Writing‐review & editing (equal).

## Supporting information

Supplementary Material

## Data Availability

Genotypes are posted on Figshare, at http://doi.org/10.6084/m9.figshare.10394711. Code is in Appendix S1.
