# Supplementary material for: Evaluating wildlife translocations using genomics: A bighorn sheep case study
Source: Ecol Evol. 2020 Nov 21;10(24):13687–704. doi: 10.1002/ece3.6942 (PMC7771163; doi:10.1002/ece3.6942)
Supplement: Supplementary file 1 — Supplementary Material [file ECE3-10-13687-s001.docx]

**Supplemental Information for:**

**Evaluating wildlife translocations using genomics: a bighorn sheep case study**

Elizabeth P. Flesch, Tabitha A. Graves, Jennifer M. Thomson, Kelly M. Proffitt,

P.J. White, Thomas R. Stephenson, Robert A. Garrott

**Table of Contents:**

| **Table S1.** Population size of evaluated herds | Page 2 |
| --- | --- |
| **Table S2.** Translocation records | Page 3 |
| **Figure S1.** fastStructure plots | Page 5 |
| **Figure S2.** Treemix variability and likelihood plots | Page 6 |
| **Figure S3.** Treemix heatmap | Page 7 |
| **Table S3.** Three-population test results | Page 7 |
| **Table S4.** Mean kinship table/heatmap of Castle Reef cluster herds | Page 8 |
| **Figure S4.** Boxplots of kinship between reintroduced herds and founding source herds | Page 9 |
| **Methods S1.** Additional methods information for fastStructure and Treemix analyses | Page 10 |
| **Results S1.** Additional information regarding analysis results | Page 12 |
| **References** | Page 14 |
| **Appendix S1.** Code used for analyses | Page 16 |

Table S1. Recent population count or estimate for evaluated bighorn sheep herds (George et al., 2009; Montana Department of Fish, Wildlife and Parks, 2010). Ecological region in Montana was defined by Montana Department of Fish, Wildlife and Parks (2010). An asterisk indicates that the value represents an aggregate of four Montana administrative units (hunting districts 421, 422, 423, and 424).

| **Herd** | **Origin** | **Year** | **Population size**  **(Estimate or Count)** | **Ecological Region in Montana** |
| --- | --- | --- | --- | --- |
| Beartooth-Absaroka | Native | 2018 | 3800 | n/a (Wyoming) |
| Castle Reef | Native | 2019 | 380* | Prairie/Mountain Foothills |
| Dinosaur | Reintroduced | 2009 | 165 | n/a (Colorado/Utah) |
| Fergus | Reintroduced | 2008 | 348 | Prairie/Breaks |
| Galton | Native | 2019 | 90 | Northwest Montane |
| Glacier | Native | 2010 | 650 | Northwest Montane |
| Highlands | Reintroduced | 2019 | 150 | Mountain Foothills |
| Lost Creek | Reintroduced | 2018 | 50 | Mountain Foothills |
| Middle Missouri | Reintroduced | 2019 | 199 | Prairie/Breaks |
| Paradise | Reintroduced | 2019 | 324 | Northwest Montane |
| Petty Creek | Reintroduced | 2018 | 142 | Northwest Montane |
| Sierra Nevada- Sawmill | Native | 2019 | 100 | n/a (California) |
| Sierra Nevada- Wheeler | Reintroduced | 2019 | 100 | n/a (California) |
| Spanish Peaks | Native | 2017 | 198 | Southern Mountains |
| Stillwater | Native | 2019 | 80 | Southern Mountains |
| Taylor Hilgard | Native | 2018 | 186 | Southern Mountains |
| Tendoys | Reintroduced | 2019 | 70 | Mountain Foothills |
| Wild Horse Island | Reintroduced | 2019 | 139 | Northwest Montane |

Table S2. All known translocations to bighorn sheep populations included in this study, including classification data when available. All source locations originated in Montana unless otherwise indicated. The herd receiving animals is generalized to the name of a population included in this study and may include multiple geographic locations. An asterisk after the total number of bighorn sheep indicates that biologists suspected the translocation failed and did not contribute to the recipient population.

| **Year** | **Herd Receiving Animals** | **Herd Providing Animals** | **Unknown Age/Sex** | **Adult**  **Male Female** | | **Lamb**  **Male Female Unknown** | | | **Total** | **Citation** |
| --- | --- | --- | --- | --- | --- | --- | --- | --- | --- | --- |
| 1970 | Beartooth-Absaroka (Wyoming Hunt Unit 5) | Whiskey Mountain (Wyoming Hunt Unit 10) | 23 |  | |  | | | 23 | (Wild Sheep Working Group, 2015) |
| 1973 | Beartooth-Absaroka (Wyoming Hunt Unit 22) | Whiskey Mountain (Wyoming Hunt Unit 10) | 17 |  | |  | | | 17 | (Wild Sheep Working Group, 2015) |
| 1944 | Castle Reef | West Gallatin |  | 1 |  |  |  |  | 1 | (Montana Department of Fish, Wildlife and Parks, 2010) |
| 1960 | Castle Reef | Castle Reef | 8 |  |  |  |  |  | 8 | (Montana Department of Fish, Wildlife and Parks, 2010) |
| 1999 | Castle Reef | E. Fork Bitterroot |  | 1 | 13 |  | 8 |  | 22 | (Montana Department of Fish, Wildlife and Parks, 2010) |
| 1952 | Dinosaur | Rifle Hogback, CO |  | 5 | 12 |  |  |  | 17 | (George et al., 2009) |
| 1952 | Dinosaur | Tarryall Range, CO |  | 3 | 12 |  |  |  | 15 | (George et al., 2009) |
| 1977 | Dinosaur | Mt. Evans, CO |  | 3 | 12 | 3 | 2 |  | 20 | (Singer & Gudorf, 1999) |
| 1983 | Dinosaur | Basalt, CO |  | 4 | 9 | 3 | 5 |  | 21 | (Singer & Gudorf, 1999) |
| 1983 | Dinosaur | Whiskey Mountain, WY |  | 5 | 10 | 1 | 3 |  | 19 | (Singer & Gudorf, 1999; Utah Division of Wildlife Resources, 2013; Wyoming Game and Fish Department, 2006) |
| 1984 | Dinosaur | Rocky Mountain National Park (Never Summers), CO |  | 1 | 13 |  |  | 5 | 19 | (George et al., 2009) |
| 1984 | Dinosaur | Whiskey Mountain, WY | 17 |  |  |  |  |  | 17 | (Utah Division of Wildlife Resources, 2013; Wyoming Game and Fish Department, 2006) |
| 1989 | Dinosaur | Whiskey Mountain, WY |  | 3 | 13 | 3 | 2 |  | 21 | (Singer & Gudorf, 1999; Utah Division of Wildlife Resources, 2013; Wyoming Game and Fish Department, 2006) |
| 1997 | Dinosaur | Dome Rock SWA, CO |  | 3 | 10 |  |  | 8 | 21 | (George et al., 2009) |
| 2000 | Dinosaur | Georgetown, CO | 8 | 6 | 7 |  |  | 6 | 27 | (George et al., 2009) |
| 1959 | Fergus | National Bison Range | 13 |  |  |  |  |  | 13 | (Montana Department of Fish, Wildlife and Parks, 2010) |
| 1960 | Fergus | National Bison Range | 11 |  |  |  |  |  | 11 | (Montana Department of Fish, Wildlife and Parks, 2010) |
| 1961 | Fergus | Castle Reef | 12 |  |  |  |  |  | 12 | (Montana Department of Fish, Wildlife and Parks, 2010) |
| 1980 | Fergus | Castle Reef | 28 |  |  |  |  |  | 28 | (Montana Department of Fish, Wildlife and Parks, 2010) |
| 1967 | Highlands | Castle Reef | 22 |  |  |  |  |  | 22 | (Montana Department of Fish, Wildlife and Parks, 2010) |
| 1969 | Highlands | Castle Reef | 31 |  |  |  |  |  | 31 | (Montana Department of Fish, Wildlife and Parks, 2010) |
| 2000 | Highlands | Castle Reef | 15 |  |  |  |  |  | 15 | (Montana Department of Fish, Wildlife and Parks, 2010) |
| 2001 | Highlands | Lower Blackfoot | 3 |  |  |  |  |  | 3 | (Montana Department of Fish, Wildlife and Parks, 2010) |
| 2001 | Highlands | Castle Reef | 17 |  |  |  |  |  | 17 | (Montana Department of Fish, Wildlife and Parks, 2010) |
| 2002 | Highlands | E. Fork Bitterroot | 14 |  |  |  |  |  | 14 | (Montana Department of Fish, Wildlife and Parks, 2010) |
| 2007 | Highlands | Ruby Mountains | 9 |  | 8 |  |  |  | 17 | (Montana Department of Fish, Wildlife and Parks, 2010) |
| 2008 | Highlands | Castle Reef |  | 5 | 57 |  |  | 3 | 65 | (Montana Department of Fish, Wildlife and Parks, 2010) |
| **Year** | **Herd Receiving Animals** | **Herd Providing Animals** | **Unknown Age/Sex** | **Adult**  **Male Female** | | **Lamb**  **Male Female Unknown** | | | **Total** | **Citation** |
| 2014 | Highlands | Fergus |  |  | 9 |  |  |  | 9 | (V. Boccadori, personal communication, November 24, 2015) |
| 1967 | Lost Creek | Castle Reef |  | 5 | 20 |  |  |  | 25 | (Montana Department of Fish, Wildlife and Parks, 2010) |
| 1985 | Lost Creek | North Clark Fork | 2 |  |  |  |  |  | 2 | (Montana Department of Fish, Wildlife and Parks, 2010) |
| 1980 | Middle Missouri | Castle Reef | 28 |  |  |  |  |  | 28 | (Montana Department of Fish, Wildlife and Parks, 2010) |
| 1979 | Paradise | Wild Horse Island |  | 5 | 9 |  |  |  | 14 | (Montana Department of Fish, Wildlife and Parks, 2010) |
| 2011 | Paradise | Wild Horse Island | 22 |  |  |  |  |  | 22 | (B. Sterling, personal communication, November 24, 2015) |
| 1968 | Petty Creek | Castle Reef |  | 1 | 10 | 2 | 3 |  | 16 | (Montana Department of Fish, Wildlife and Parks, 2010) |
| 1985 | Petty Creek | National Bison Range |  | 4 |  |  |  |  | 4 | (Montana Department of Fish, Wildlife and Parks, 2010) |
| 1979 | Sierra Nevada: Wheeler Ridge | Sierra Nevada, CA: Sawmill/Baxter |  | 4 | 3 | 1 | 1 |  | 9 | (Wild Sheep Working Group, 2015) |
| 1980 | Sierra Nevada: Wheeler Ridge | Sierra Nevada, CA: Mt. Baxter |  | 1 | 7 | 1 | 1 |  | 10 | (Wild Sheep Working Group, 2015) |
| 1982 | Sierra Nevada: Wheeler Ridge | Sierra Nevada, CA: Mt. Baxter |  | 4 |  |  |  |  | 4 | (Wild Sheep Working Group, 2015) |
| 1986 | Sierra Nevada: Wheeler Ridge | Sierra Nevada, CA: Sawmill/Baxter |  | 1 | 3 |  |  |  | 4 | (Wild Sheep Working Group, 2015) |
| 2005 | Sierra Nevada: Sawmill Canyon | Sierra Nevada, CA: Wheeler |  |  | 3 |  |  |  | 3 | (T. Stephenson, personal communication, March 9, 2019) |
| 1947 | Spanish Peaks | Kootenai Falls |  | 2 |  |  |  |  | 2 | (Montana Department of Fish, Wildlife and Parks, 2010) |
| 1974 | Spanish Peaks | Castle Reef |  | 2 |  |  |  |  | 2 | (Montana Department of Fish, Wildlife and Parks, 2010) |
| 1970 | Stillwater | Castle Reef |  | 2 |  |  |  |  | 2* | (Montana Department of Fish, Wildlife and Parks, 2010) |
| 1984 | Stillwater | National Bison Range |  | 3 |  |  |  |  | 3* | (Montana Department of Fish, Wildlife and Parks, 2010) |
| 1988 | Taylor Hilgard | North Clark Fork |  | 2 | 11 |  |  | 6 | 19 | (Montana Department of Fish, Wildlife and Parks, 2010; Roy & Irby, 1994) |
| 1989 | Taylor Hilgard | North Clark Fork | 5 |  |  |  |  |  | 5 | (Montana Department of Fish, Wildlife and Parks, 2010) |
| 1989 | Taylor Hilgard | Lost Creek | 18 |  |  |  |  |  | 18 | (Montana Department of Fish, Wildlife and Parks, 2010) |
| 1993 | Taylor Hilgard | Wild Horse Island | 26 |  |  |  |  |  | 26 | (Montana Department of Fish, Wildlife and Parks, 2010) |
| 1984 | Tendoys | Lost Creek |  | 13 | 26 |  |  |  | 39 | (Montana Department of Fish, Wildlife and Parks, 2010) |
| 1986 | Tendoys | North Clark Fork |  | 13 | 1 |  |  |  | 14 | (Montana Department of Fish, Wildlife and Parks, 2010) |
| 1997 | Tendoys | John Long Range |  | 5 | 15 |  |  |  | 20* | (Montana Department of Fish, Wildlife and Parks, 2010) |
| 2002 | Tendoys | Castle Reef |  | 3 | 27 |  |  |  | 30 | (Montana Department of Fish, Wildlife and Parks, 2010) |
| 2012 | Tendoys | Wild Horse Island |  | 5 | 40 |  |  | 4 | 49 | (B. Sterling, personal communication, November 24, 2015) |
| 1939 | Wild Horse Island | Mission Range |  | 1 | 1 |  |  |  | 2 | (Montana Department of Fish, Wildlife and Parks, 2010) |
| 1947 | Wild Horse Island | Castle Reef | 6 |  |  |  |  |  | 6 | (Montana Department of Fish, Wildlife and Parks, 2010) |
| 1987 | Wild Horse Island | Ural-Tweed |  | 2 |  |  |  |  | 2 | (Montana Department of Fish, Wildlife and Parks, 2010) |

Figure S1. Results from fastStructure major modes, including the most supported (K=6) shown in text, K=7, and K=9, aligned using CLUMPAK (Kopelman et al., 2015; Raj et al., 2014). Major mode K=6 had support of 86/100 replicates, K=7 had support of 72/100, and K=9 had support of 48/100.


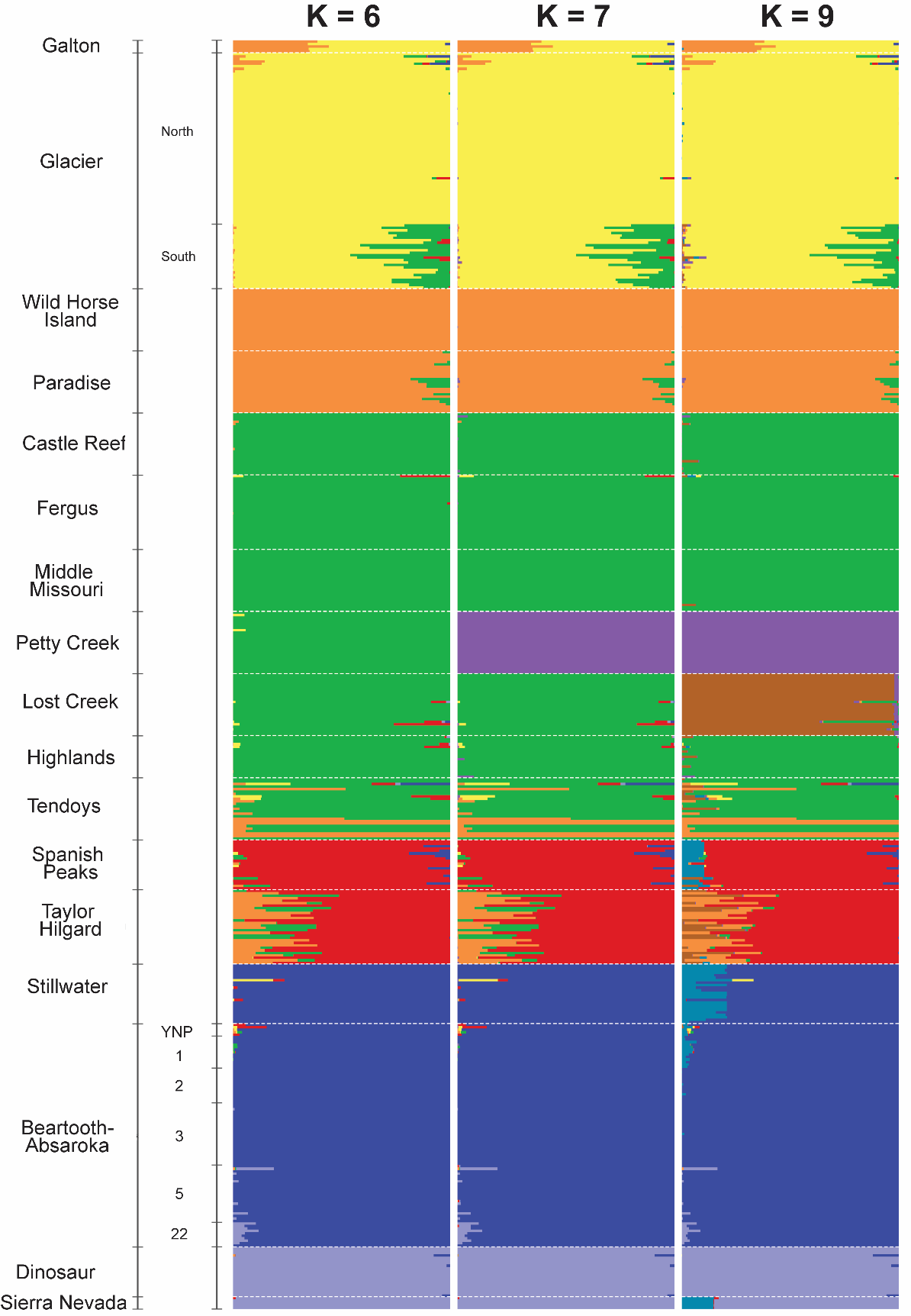


Figure S2. Treemix model variability explained (A) and likelihood (B) for 50 replicates per migration value.


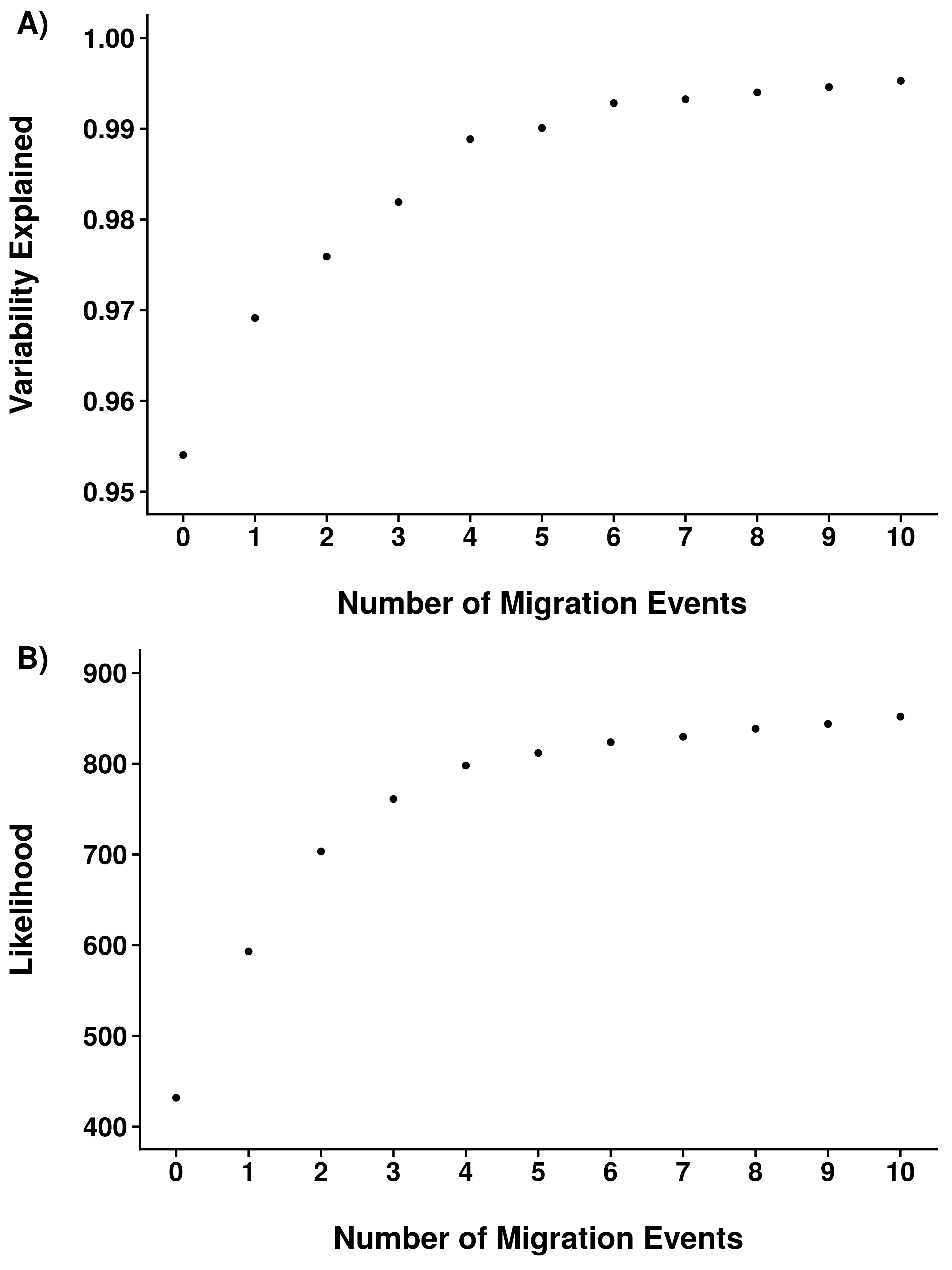


Figure S3. Treemix residuals from the four migrations model for herds with a sample size of at least 20 bighorn sheep. Residuals greater than zero (greens, blues, black) represent herds that the software identified as populations that are more related to one another than shown in the tree, meaning there is a possibility for past admixture events.


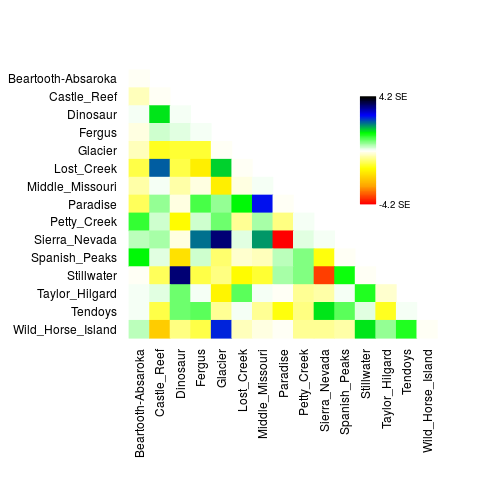


Table S3. Results of three-population test for all herds included in the Treemix analysis (Pickrell & Pritchard, 2012; Reich et al., 2009). Only negative *f_3_* statistics are shown. A negative value of the *f_3_* statistic suggests that the herd listed as “Population A” is admixed.
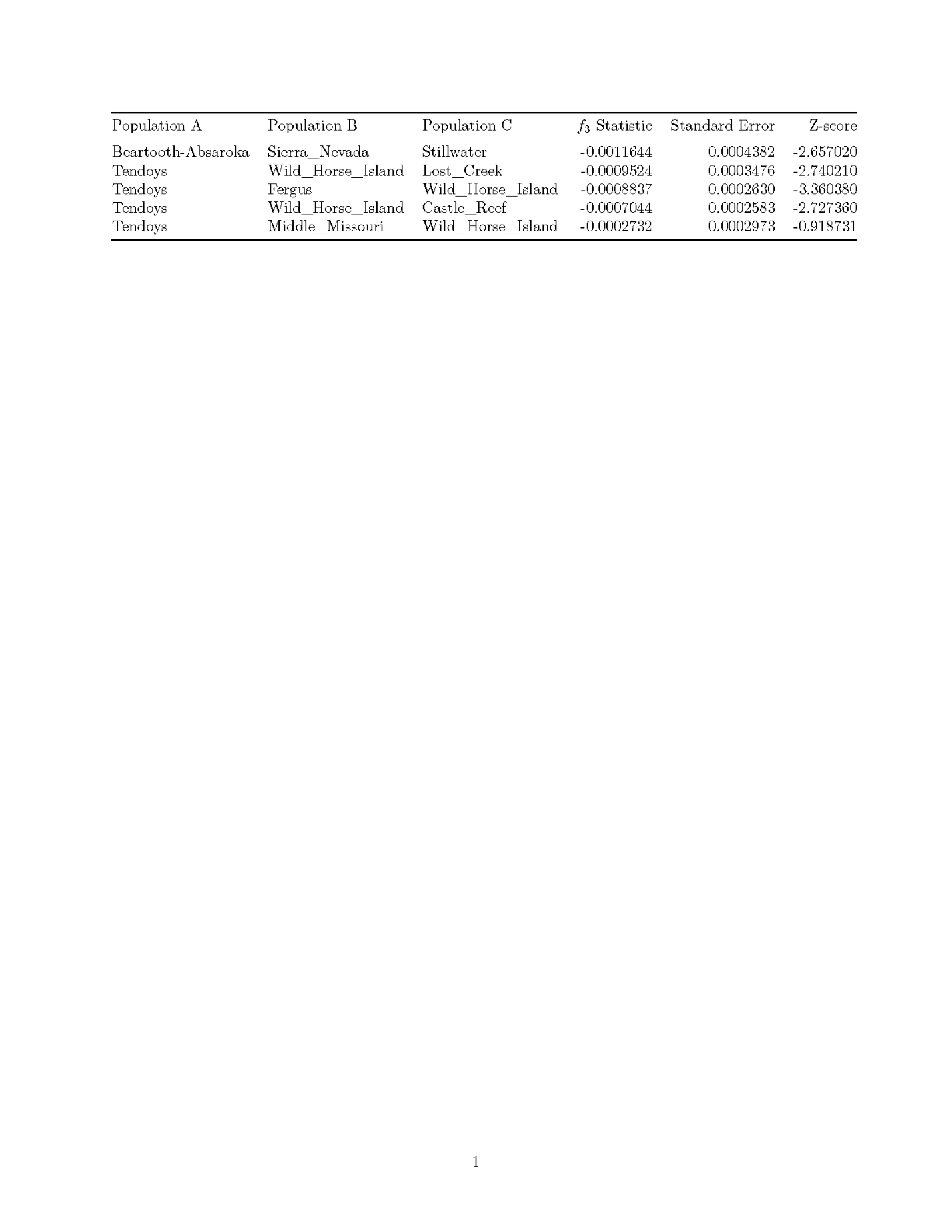


Table S4. Mean kinship of all herds with the same fastStructure cluster as Castle Reef (K=6), to demonstrate translocation options for wildlife managers to maximize genetic diversity. Standard deviation from the mean is in parentheses. Smaller values indicate lower mean kinship. Sample size from each population is shown to the right of herd name; herds with less than 20 samples are labelled in red. For example, to select an augmentation source for Middle Missouri, we would consider mean kinship values with herds in the same fastStructure cluster: Castle Reef (-0.001), Fergus (-0.035), Tendoys (-0.041), Highlands (-0.058), Lost Creek (-0.101) and Petty Creek (-0.126). Based on mean kinship, Petty Creek and Lost Creek would be optimal sources to maximize genetic diversity, as these populations have low mean kinship with Middle Missouri.


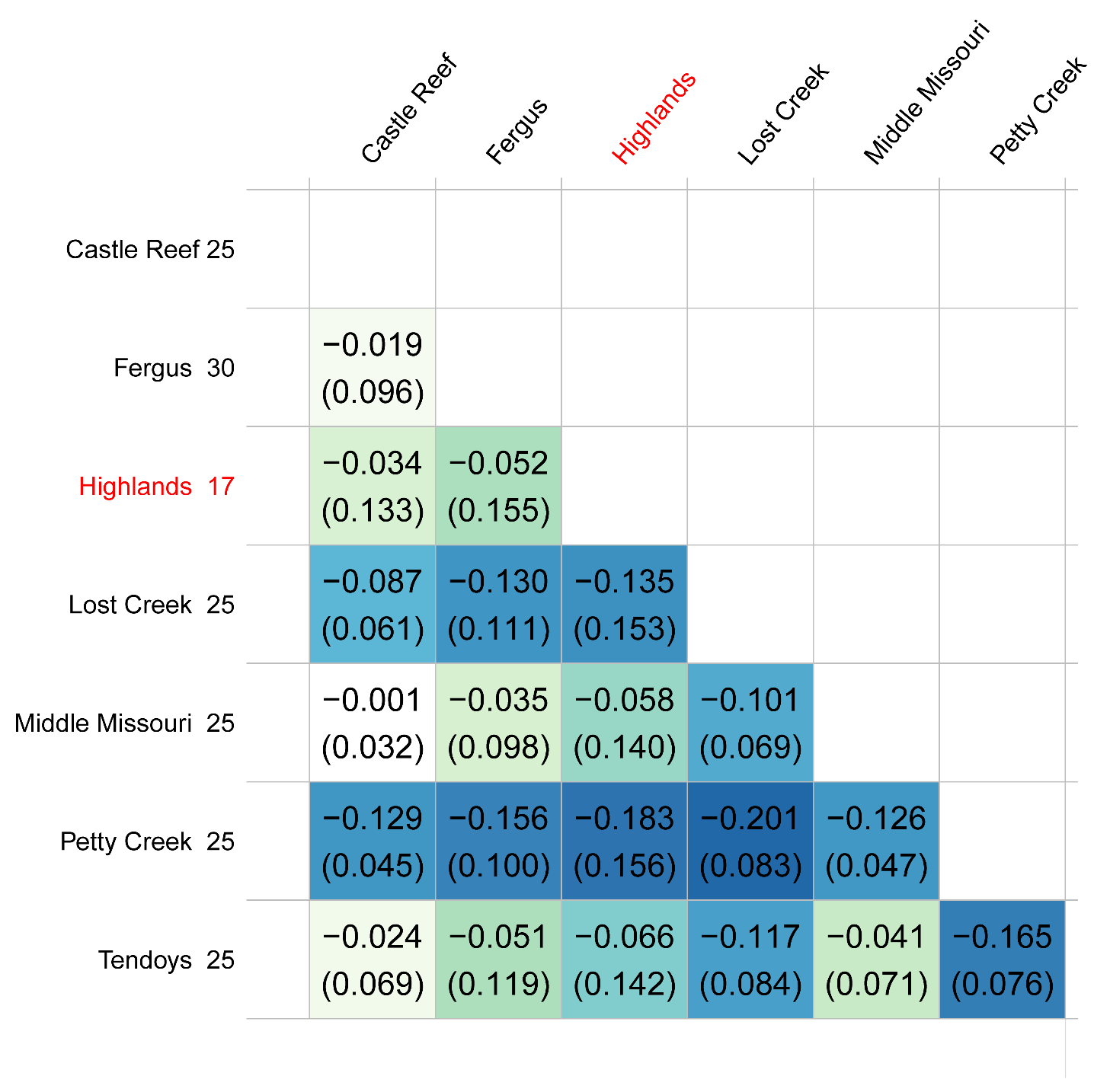


Figure S4. Boxplots of kinship for reintroduced herds with their founding source, in comparison to herd reintroduction information (A, B), augmentation records (C, D, E), and potential genetic connectivity with other herds (F).


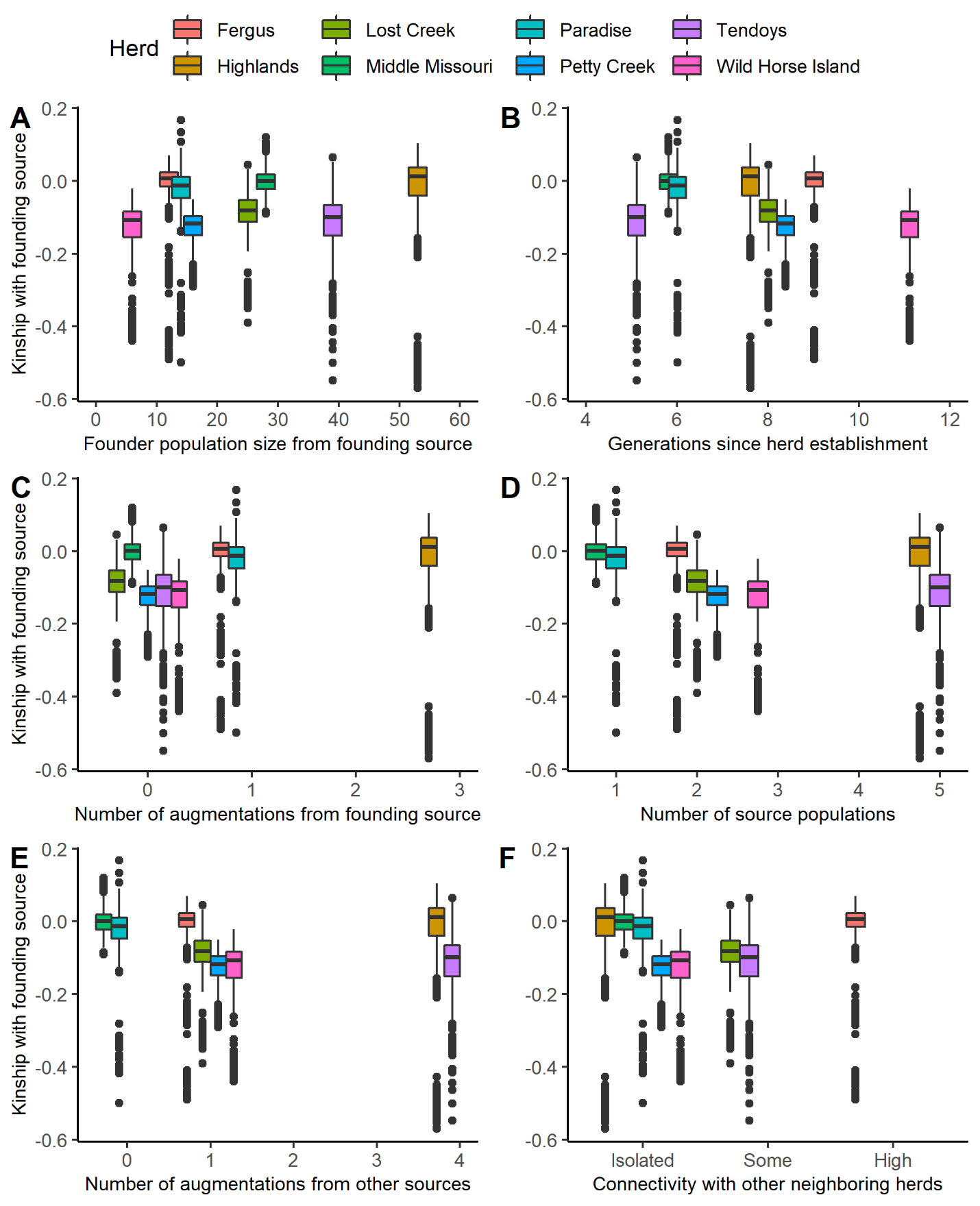


**Methods S1**

*fastStructure*

To estimate global ancestry and assess the number of populations (K clusters) in our dataset, we used a variational Bayesian framework to approximate population structure for genomic datasets (Pritchard et al., 2000; Raj et al., 2014). This model was implemented in fastStructure v1.0 through structure_threader v1.2.15 (Pina‐Martins et al., 2017; Raj et al., 2014). The program structure_threader assists with running multiple replicates and evaluating the number of clusters that maximize marginal likelihood of the model (Pina‐Martins et al., 2017; Raj et al., 2014). First, we ran 10 replicates using K values of 1 to 17 and a simple prior model with fivefold validation and randomized seed selection. We used the chooseK.py script from the fastStructure repository to evaluate the marginal likelihood of K values and determine which K values should be evaluated with additional replicates (Raj et al., 2014). Six out of 10 replicates identified K=6 as the model complexity that maximized marginal likelihood, and the remaining replicates identified K=7. For the number of model components used to explain subtle structure in the data, 9 out of 10 replicates identified K values between 8 and 11. Thus, we evaluated K values of 6 to 11 using 100 replicates with a simple prior, fivefold validation, and randomized seed selection.

We used CLUMPAK to realign clusters and generate consensuses across multiple runs (Kopelman et al., 2015). We uploaded the results to the CLUMPAK online server to realign clusters and generate consensuses among replicates (Kopelman et al., 2015). We used the online server default settings: LargeKGreedy algorithm, random input order, and 2,000 repeats. We created bar plots from the CLUMPAK consensus files using R package pophelper 2.2.7 (Francis, 2017; Kopelman et al., 2015; R Core Team, 2017). We summed all cluster assignments by population to generate a herd-level pie chart of fastStructure results and mapped the pie charts using ArcGIS (ESRI (Environmental Systems Resource Institute), 2010).

*Multidimensional scaling*

To identify clusters of samples without relying on assumptions regarding the cause of population structure or Hardy-Weinberg equilibrium, we completed a nested multidimensional scaling (MDS) analysis using KING v2.1.4 (Manichaikul et al., 2010). MDS is an unsupervised analysis that reduced the dimensionality of the genomic dataset to plot relationships among individual samples (Pevsner, 2015). MDS can be more accurate than a principle components analysis in discerning differences among samples using genomic data, but does not report the percent of variance explained by each axis (Pevsner, 2015). KING employs classical MDS using Euclidean distance, where each SNP is not standardized by the standard deviation. We first completed an MDS of the entire dataset to visually identify predominant clusters of samples. Next, we analyzed each cluster of samples found in the first two dimensions of the overall plot, again using MDS to understand differences within the higher order clusters.

*Treemix*

To describe temporal patterns of ancestry, we used Treemix v1.13 for analysis and plotting of a maximum likelihood tree of populations (Pickrell & Pritchard, 2012). Treemix used allele frequencies from each population and Gaussian approximation for genetic drift to estimate a tree with each population on a branch (Pickrell & Pritchard, 2012). Possible admixture events between branches of the tree were evaluated using a stepwise likelihood approach, where the software searched the tree for the optimal location of each migration event (Pickrell & Pritchard, 2012). We evaluated only Rocky Mountain bighorn sheep populations that had a sample size of at least 20 to avoid population allele frequency bias (Flesch et al., 2018; Günther et al., 2016). We identified Sierra Nevada as the root of the tree. We relabeled “migration” weight and order as “translocation,” as all detected gene flow events were due to known translocation efforts (Table S1).

To ensure that the block size set for the Treemix analysis was large enough to avoid linkage disequilibrium (LD) among SNPs, we completed an LD decay analysis using PLINK (Purcell et al., 2007). We used the r^2^ statistic, which calculated the squared correlations among SNPs based on genotype allele counts (Purcell et al., 2007). Due to prior LD pruning and a low density of SNPs after filtering, the LD decay analysis indicated that a block size of 10 SNPs was acceptable for use in the Treemix analysis. We used the “global” option in Treemix to complete global rearrangements after all populations were added. We generated 50 replicates of population trees for each value of migration, ranging from 0 to 10 possible migrations. For each migration value, we evaluated residuals as a heatmap to evaluate the possibility for additional admixture events between populations not shown in the tree. We evaluated the variability explained by the model and model likelihood to determine the optimal number of migration events (Figure S2).

To further evaluate the population tree with the identified optimal number of migration events, we completed a bootstrap analysis with 1,000 replicates using BITE v1.2.0003 (Milanesi et al., 2017). BITE assisted in implementation of bootstrap replicates in Treemix and built a consensus tree of replicates using majority rule and the *consense* executable from PHYLIP v3.697 (Felsenstein, 1989, 2005; Milanesi et al., 2017). We evaluated the consensus tree that displayed bootstrap support and the output files from PHYLIP to determine bootstrap support for each node. Finally, we completed a three-population test for treeness using threepop 0.1 in Treemix (Pickrell & Pritchard, 2012; Reich et al., 2009). The three-population test generated f-statistics that aided in evaluating the population tree and migrations. A negative value of the *f_3_* statistic suggested that a specific population was admixed from groups related to two other identified populations, whereas a positive value indicated that the three considered populations were simply related to a common ancestor in a bifurcating tree without admixture (Pickrell & Pritchard, 2012; Reich et al., 2009).

*Kinship*

We estimated mean kinship between populations using KING v2.1.4, because kinship is a useful metric for genetic management (Ballou & Lacy, 1995; Manichaikul et al., 2010). Kinship, also called coancestry, is the probability that two randomly sampled alleles from two individuals are identical by descent (Manichaikul et al., 2010). Mean kinship calculated between populations serves as a measure of population similarity, with higher values interpreted as populations that are more related (Frankham et al., 2017). To assess how characteristics of translocations influenced the current kinship between reintroduced populations and their founding source, we evaluated several herd attributes that may affect divergence of reintroduced populations using boxplots. Unassisted gene flow from neighboring populations was evaluated using previous research that evaluated GPS data (T. Graves unpublished data; Lowrey et al., 2020; Lula et al., 2020) and by asking area biologists for their opinion on the level of connectivity with other bighorn sheep populations, ranging from “isolated” (no gene flow expected), “some,” and “high” (exchange with neighboring populations likely).

**Results S1**

*Genomic dataset and quality control*

To filter the dataset for analysis, we first excluded the X chromosome and SNPs with unknown mappings using Golden Helix software, which removed 29,411 SNPs and resulted in 576,595 remaining SNPs (*SNP & Variation Suite*, 2016). We exported the remaining SNP data and positions (.ped and .map formats) to complete the remaining filtering in PLINK (Purcell et al., 2007). Filtering SNPs using a minor allele frequency of less than 0.0001 removed 468,976 SNPs, which means that these SNPs were monomorphic in the examined bighorn sheep. Applying a SNP call rate requirement of 0.99 removed 74,330 SNPs and resulted in 33,289 SNPs remaining, which were used for kinship calculations. To infer population structure and ancestry, we further filtered the dataset. Applying a minor allele frequency of 0.01 removed 25,206 SNPs. Requiring a Hardy-Weinberg equilibrium p-value of less than 0.00001 removed 1,654 SNPs. We completed linkage disequilibrium (LD) pruning using a window size of 100, window increment of 25, and LD statistic of r^2^, which removed 274 SNPs and resulted in 6,155 SNPs remaining at a density of one SNP per 399.585kb. We completed the fastStructure, MDS, and Treemix analyses using the dataset of 6,155 SNPs.

*fastStructure*

We completed fastStructure analyses of K values ranging from 6 to 11 using 100 replicates each, and 77 out of 100 runs identified K=6 as the model complexity that maximized marginal likelihood. After aligning all runs using CLUMPAK, the major mode for K=6 had support of 86 out of 100 runs, which was the greatest number of runs supporting the major mode out of all evaluated K values. Nineteen out of 100 runs identified K=7 as the model complexity that maximized marginal likelihood; 4 out of 100 runs identified K=8 as the model complexity that maximized marginal likelihood. Thus, we discuss the K=6 major mode as our main fastStructure result (Figure 1).

*Treemix*

We evaluated our Treemix analyses using plots of explained variability and model likelihood (Figure S2). The explained variability (Figure S2A) and likelihood (Figure S2B) were the same for all 50 replicates within each migration event value. Over 95% of the variability in the dataset was explained by the tree structure with no migrations, which may explain why there was not variation among the individual replicates per migration value. The plots of explained variability and likelihood suggested the optimal population tree was the model with four migration events. This decision was further supported by the fact that we had no record of the translocation plotted for migration event #5. There was 100% bootstrap support for all nodes in the tree with four migrations plotted, which was likely due to about 99% of variability being explained by the model (Figure S2A). We plotted the residuals for the model with four migration events (Figure S3), and residuals greater than zero represented pairs of populations that may be more related to one another than shown in the tree, meaning there were potentially past admixture events (Pickrell & Pritchard, 2012). Multiple residuals were greater than zero, rather than a single residual much greater than the others, lending further support to the population tree with four migration events. Thus, we plotted the population tree for one Treemix replicate with four migration events (Figure 3).

*Mean kinship*

We estimated mean kinship between reintroduced herds and their founding source and evaluated six attributes that could affect this kinship and population evolution since reintroduction (Figure S4). Founder population size and initial allele frequencies in a reintroduced herd provided the variation available for selection and genetic drift to act over time, so we evaluated founder population size and the number of generations since herd establishment (Frankham et al., 2017). However, there was no clear pattern of a relationship between these two attributes and kinship with the founding population (Figure S4A; Figure S4B). These relationships were often complicated by other translocations, and we expected that additional augmentations from the founding source would result in greater mean kinship. Populations with multiple or supplemental augmentations from their founding source, including Highlands, Paradise, and Fergus, generally had higher mean kinships than those that did not (Figure S4C).

In addition, we expected that augmented gene flow from other populations would result in lower mean kinship with the founding source. Thus, we evaluated the total number of augmentations and source populations translocated to the reintroduced herd, as well as natural connectivity with herds in geographic proximity. As the number of augmentations from other areas and the number of source populations increased, mean kinship with the founding source generally decreased (Figure S4D; Figure S4E). Highlands was an exception, with five source populations and four augmentations from other sources. High mean kinship between the Highlands and its founding source, Castle Reef, suggested that augmentations from other areas, including multiple herds not in this study, may have failed to make a genetic contribution.

We expected that unassisted gene flow would result in lower mean kinship with the founding source, but this question was generally difficult to evaluate with our low sample size for the “some” and “high” connectivity categories (Figure S4F). Fergus had high connectivity but high mean kinship with its founding source; this was likely because the geographically proximate populations had the same founding source. In general, all six examined herd attributes likely influenced the evolution of reintroduced herds to differing extents, which complicated our interpretation of which attributes were dominant in influencing the genetics of reintroduced herds. However, applying this approach with a greater sample size could serve as a method to evaluate which population attributes influenced evolution and divergence from the founding source after reintroduction.

**References**

Ballou, J. D., & Lacy, R. C. (1995). Identifying genetically important individuals for management of genetic variation in pedigreed populations. In J. D. Ballou, M. Gilpin, & T. J. Foose (Eds.), *Population management for survival and recovery: Analytical methods and strategies in small population conservation* (pp. 76–111). Columbia University Press.

ESRI (Environmental Systems Resource Institute). (2010). *ArcGIS 10.1*.

Felsenstein, J. (1989). PHYLIP-phylogeny inference package (version 3.2). *Cladistics*, *5*, 164–166.

Felsenstein, J. (2005). *PHYLIP (Phylogeny Inference Package) version 3.6*. Department of Genome Sciences.

Flesch, E. P., Rotella, J. J., Thomson, J. M., Graves, T. A., & Garrott, R. A. (2018). Evaluating sample size to estimate genetic management metrics in the genomics era. *Molecular Ecology Resources*, *18*(5), 1077– 1091. https://doi.org/10.1111/1755-0998.12898

Francis, R. M. (2017). pophelper: An R package and web app to analyse and visualize population structure. *Molecular Ecology Resources*, *17*(1), 27–32. https://doi.org/10.1111/1755-0998.12509

Frankham, R., Ballou, J. D., Ralls, K., Eldridge, M. D. B., Dubash, M., Fenster, C. B., Lacy, R. C., & Sunnucks, P. (2017). *Genetic management of fragmented animal and plant populations*. Oxford University Press.

George, J. L., Kahn, R., Miller, M. W., & Watkins, B. (2009). *Colorado bighorn sheep management plan 2009-2019* (No. 81; p. 93). Colorado Division of Wildlife.

Günther, T., Lampei, C., Barilar, I., & Schmid, K. J. (2016). Genomic and phenotypic differentiation of Arabidopsis thaliana along altitudinal gradients in the North Italian Alps. *Molecular Ecology*, *25*(15), 3574–3592. https://doi.org/10.1111/mec.13705

Kopelman, N. M., Mayzel, J., Jakobsson, M., Rosenberg, N. A., & Mayrose, I. (2015). Clumpak: A program for identifying clustering modes and packaging population structure inferences across K. *Molecular Ecology Resources*, *15*(5), 1179–1191. https://doi.org/10.1111/1755-0998.12387

Lowrey, B., McWhirter, D. E., Proffitt, K. M., Monteith, K. L., Courtemanch, A. B., White, P. J., Paterson, J. T., Dewey, S. R., & Garrott, R. A. (2020). Individual variation creates diverse migratory portfolios in native populations of a mountain ungulate. *Ecological Applications*, *30*(5). https://doi.org/10.1002/eap.2106

Lula, E. S., Lowrey, B., Proffitt, K. M., Litt, A. R., Cunningham, J. A., Butler, C. J., & Garrott, R. A. (2020). Is Habitat Constraining Bighorn Sheep Restoration? A Case Study. *The Journal of Wildlife Management*, *84*(3), 588–600. https://doi.org/10.1002/jwmg.21823

Manichaikul, A., Mychaleckyj, J. C., Rich, S. S., Daly, K., Sale, M., & Chen, W.-M. (2010). Robust relationship inference in genome-wide association studies. *Bioinformatics*, *26*(22), 2867–2873. https://doi.org/10.1093/bioinformatics/btq559

Milanesi, M., Capomaccio, S., Vajana, E., Bomba, L., Garcia, J. F., Ajmone-Marsan, P., & Colli, L. (2017). BITE: An R package for biodiversity analyses. *BioRxiv*, 181610. https://doi.org/10.1101/181610

Montana Department of Fish, Wildlife and Parks. (2010). *Montana bighorn sheep conservation strategy*. Montana Department of Fish, Wildlife and Parks.

Pevsner, J. (2015). *Bioinformatics and Functional Genomics* (Third). John Wiley & Sons.

Pickrell, J. K., & Pritchard, J. K. (2012). Inference of population splits and mixtures from genome-wide allele frequency data. *PLoS Genetics*, *8*(11), e1002967. https://doi.org/10.1371/journal.pgen.1002967

Pina‐Martins, F., Silva, D. N., Fino, J., & Paulo, O. S. (2017). Structure_threader: An improved method for automation and parallelization of programs structure, fastStructure and MavericK on multicore CPU systems. *Molecular Ecology Resources*, *17*(6), 268–274. https://doi.org/10.1111/1755-0998.12702

Pritchard, J. K., Stephens, M., & Donnelly, P. (2000). Inference of population structure using multilocus genotype data. *Genetics*, *155*(2), 945–959.

Purcell, S., Neale, B., Todd-Brown, K., Thomas, L., Ferreira, M. A. R., Bender, D., Maller, J., Sklar, P., de Bakker, P. I. W., Daly, M. J., & Sham, P. C. (2007). PLINK: A tool set for whole-genome association and population-based linkage analyses. *The American Journal of Human Genetics*, *81*(3), 559–575. https://doi.org/10.1086/519795

R Core Team. (2017). *R: A language and environment for statistical computing*. R Foundation for Statistical Computing. https://www.R-project.org/

Raj, A., Stephens, M., & Pritchard, J. K. (2014). FastSTRUCTURE: Variational inference of population structure in large SNP data sets. *Genetics*, *197*, 573–589.

Reich, D., Thangaraj, K., Patterson, N., Price, A. L., & Singh, L. (2009). Reconstructing Indian population history. *Nature*, *461*(7263), 489–494. https://doi.org/10.1038/nature08365

Roy, J. L., & Irby, L. R. (1994). Augmentation of a bighorn sheep herd in southwest Montana. *Wildlife Society Bulletin (1973-2006)*, *22*(3), 470–478.

Singer, F. J., & Gudorf, M. A. (1999). *Restoration of bighorn sheep metapopulations in and near 15 national parks: Conservation of a severely fragmented species* (U.S. Geological Survey Open File Report No. 99–102; p. 96). Midcontinent Ecological Science Center.

*SNP & Variation Suite* (8.6.0). (2016). [Computer software]. Golden Helix, Inc. http://www.goldenhelix.com

Utah Division of Wildlife Resources. (2013). *Utah bighorn sheep statewide management plan*.

Wild Sheep Working Group. (2015). *Records of wild sheep translocations: United States and Canada, 1922-present*. Western Association of Fish and Wildlife Agencies.

Wyoming Game and Fish Department. (2006). *A comprehensive bighorn sheep management plan*.

**Appendix S1. Bash and R code used for analyses**

**Table of contents**

- General user information
- Filtering
- Kinship
- MDS
- fastStructure
- Treemix

**General user information**

We completed all analyses using R Markdown scripts in RStudio on a computer system running Ubuntu 16.04.5 LTS, codename xenial. Software requirements are listed in each section; see manuscript text for software citations. Software listed frequently require other dependency programs to be installed separately; see software help manuals.

File names assume a project folder set up with the following folders: data, figures, manuscript, output, and scripts. The data file used in this example is entitled “bhs_all_table437” that is in PLINK format to start (.ped, .fam, and .map files). X and 99 (unmapped) chromosomes have already been deactivated. To modify this script for another dataset, search and replace “bhs_all_table437” with the name of the dataset.

**Filtering**

Software requirements:

- PLINK v1.90b4.6 64-bit (15 Aug 2017)

**Generate files for analysis and define populations**

*# Data file: bhs_all_table437- X and 99 chromosomes deactivated, 576595 SNPs, 511 sheep*

*# Generate binary and family file for input data - make sure this only occurs once*
plink --sheep --file ../data/bhs_all_table437 --out ../output/1_bhs_all_table437 --make-bed

*#Count number of SNPs and save in txt file*
plink --sheep --bfile ../output/1_bhs_all_table437 --freq counts --out ../output/1_bhs_all_table437_freq
awk 'END { print NR - 1 }' **<**../output/1_bhs_all_table437_freq.frq.counts**>** ../output/1_bhs_all_table437_marker_count.txt

*# Generate .phe file from family file to define populations*
awk ' { print $1 "\t" $2 "\t" $1} ' **<**../output/1_bhs_all_table437.fam**>** ../output/1_bhs_all_table437.phe

**Filtering for kinship calculations**

*# 1) MAF < 0.0001 removed for all samples included*
plink --sheep --bfile ../output/1_bhs_all_table437 --maf 0.0001 --allow-no-sex --out ../output/1_bhs_all_table437_1 --make-bed

*#Count number of SNPs and save in txt file*
plink --sheep --bfile ../output/1_bhs_all_table437_1 --freq counts --out ../output/1_bhs_all_table437_1_freq
awk 'END { print NR - 1 }' **<**../output/1_bhs_all_table437_1_freq.frq.counts**>** ../output/1_bhs_all_table437_1_marker_count.txt

*# 2) SNP call rate > 0.99*
plink --sheep --bfile ../output/1_bhs_all_table437_1 --geno 0.01 --allow-no-sex --out ../output/1_bhs_all_table437_2 --make-bed

*#Count number of SNPs and save in txt file*
plink --sheep --bfile ../output/1_bhs_all_table437_2 --freq counts --out ../output/1_bhs_all_table437_2_freq
awk 'END { print NR - 1 }' **<**../output/1_bhs_all_table437_2_freq.frq.counts**>** ../output/1_bhs_all_table437_2_marker_count.txt

*# Use 1_bhs_all_table437_2 for kinship calculations*

**Filtering for PCA, MDS, fastStructure, and Treemix analyses**

*# 3) MAF < 0.01 removed for all samples included*
plink --sheep --bfile ../output/1_bhs_all_table437_2 --maf 0.01 --allow-no-sex --out ../output/1_bhs_all_table437_3 --make-bed

*#Count number of SNPs and save in txt file*
plink --sheep --bfile ../output/1_bhs_all_table437_3 --freq counts --out ../output/1_bhs_all_table437_3_freq
awk 'END { print NR - 1 }' **<**../output/1_bhs_all_table437_3_freq.frq.counts**>** ../output/1_bhs_all_table437_3_marker_count.txt

*# 4) Deviate from HWE p<0.00001*
plink --sheep --bfile ../output/1_bhs_all_table437_3 --hwe 0.00001 midp --allow-no-sex --out ../output/1_bhs_all_table437_4 --make-bed

*#Count number of SNPs and save in txt file*
plink --sheep --bfile ../output/1_bhs_all_table437_4 --freq counts --out ../output/1_bhs_all_table437_4_freq
awk 'END { print NR - 1 }' **<**../output/1_bhs_all_table437_4_freq.frq.counts**>** ../output/1_bhs_all_table437_4_marker_count.txt

*# 5) LD pruning- window size 100, window increment 25, r2 threshold 0.99*
*# Huisman et al. 2016 LD pruning*
plink --sheep --bfile ../output/1_bhs_all_table437_4 --indep-pairwise 100 25 0.99 -allow-no-sex --out ../output/1_bhs_all_table437_5_prune

*#Create new bed file excluding LD pruned SNPs*
plink --sheep --bfile ../output/1_bhs_all_table437_4 --exclude ../output/1_bhs_all_table437_5_prune.prune.out --recode --out ../output/1_bhs_all_table437_5 --make-bed

*#Count number of SNPs and save in txt file*
plink --sheep --bfile ../output/1_bhs_all_table437_5 --freq counts --out ../output/1_bhs_all_table437_5_freq
awk 'END { print NR - 1 }' **<**../output/1_bhs_all_table437_5_freq.frq.counts**>** ../output/1_bhs_all_table437_5_marker_count.txt

**Kinship**

Software requirements:

- PLINK v1.90b4.6 64-bit (15 Aug 2017)
- KING 2.1.4

*# Use 1_bhs_all_table437_2.bed file based on filtering*

*# Modify SNPs on chromosomes 23-26 so that KING recognizes them (KING only recognizes chr 1-22)*
*## Add 1,000,000,000 to these positions in plink and then run KING*
*### Pull out rows that are chr 23-26*
awk '$1 >= 23 { print $0 }' ../data/bhs_all_table437.map **>** ../output/2_relationship_inference/bhs_all_table437_map_chr23to26.txt

*### Update chr 23-26 SNPs with different values to differentiate from other SNPs after chromosomes updated*
awk '{$4 = $4 + 1000000000; print $2, $4}' ../output/2_relationship_inference/bhs_all_table437_map_chr23to26.txt **>** ../output/2_relationship_inference/bhs_all_table437_map_chr23to26_2.txt

*### Update chromosomes with different values- renumber 23-26 to 13-16*
awk '{$1 = $1 - 10; print $2, $1}' ../output/2_relationship_inference/bhs_all_table437_map_chr23to26.txt **>** ../output/2_relationship_inference/bhs_all_table437_map_chr23to26_3.txt

*# Update file with new map build*
*## Add new SNP positions*
plink --sheep --bfile ../output/1_bhs_all_table437_2 --update-map ../output/2_relationship_inference/bhs_all_table437_map_chr23to26_2.txt --make-bed --out ../output/2_relationship_inference/bhs_all_table437_2_newmap1

*## Add new chromosome numbers*
plink --sheep --bfile ../output/2_relationship_inference/bhs_all_table437_2_newmap1 --update-chr ../output/2_relationship_inference/bhs_all_table437_map_chr23to26_3.txt --make-bed --out ../output/2_relationship_inference/bhs_all_table437_2_newmap2

Kinship using all SNPs, as SNPs on additional sheep chromosomes have been renumbered

*# Kinship estimate*
king -b ../output/2_relationship_inference/bhs_all_table437_2_newmap2.bed --kinship --cpus 7 --prefix ../output/2_relationship_inference/bhs_all_table437_2_newmap2_kinship

**MDS**

Software requirements:

- PLINK v1.90b4.6 64-bit (15 Aug 2017)
- KING 2.1.4

**Estimate with LD pruned dataset**

Use dataset with map that will use all SNPs in KING, as in Kinship section

*# Update file with new map build*
*## Add new SNP positions*
plink --sheep --bfile ../output/1_bhs_all_table437_5 --update-map ../output/2_relationship_inference/bhs_all_table437_map_chr23to26_2.txt --make-bed --out ../output/3_pca/1_bhs_all_table437_5_newmap1

*## Add new chromosome numbers*
plink --sheep --bfile ../output/3_pca/1_bhs_all_table437_5_newmap1 --update-chr ../output/2_relationship_inference/bhs_all_table437_map_chr23to26_3.txt --make-bed --out ../output/3_pca/bhs_all_table437_5_newmap2

*# MDS analysis*
king -b ../output/3_pca/bhs_all_table437_5_newmap2.bed --mds --prefix ../output/3_pca/bhs_all_table437_5_newmap2_mds

**fastStructure**

Software requirements:

- fastStructure
- structure_threader
- python3
- CLUMPAK (online server used)
- pophelper (R package used for plotting)

**Create popfile (populations and order) for input into structure_threader**

*# Create list of areas to label samples from fam file*
*## Count number of observations per herd, sort alphabetically, and add row number*
cat ../output/1_bhs_all_table437_5.fam **|** awk '{arr[$1]++}END{for (a in arr) print a, arr[a]}' **|** sort **|** awk '{print $1, $2, NR}'**>** ../output/6a_structure_threader/1_bhs_all_table437_5_popfile.txt

**RUN STRUCTURE_THREADER: SIMPLE PRIOR- full dataset 10 replicates, 5 fold validation- 1 hour per replicate**

**1) All data- 10 replicates to determine K values to examine with 100 replicates**

*# Create directory for output*
mkdir -p ../output/6a_structure_threader/1_all_K1to17_10reps_full

*# Generate random seed*
**for** i in {1..10}
**do**
SEED=$RANDOM

*# Run program and use fivefold validation with 8 threads*
structure_threader run -K 17 -i ../output/1_bhs_all_table437_5.bed -o ../output/6a_structure_threader/1_all_K1to17_10reps_full/1_bhs_all_table437_5_${i} --pop ../output/6a_structure_threader/1_bhs_all_table437_5.txt -t 8 -fs /usr/local/bin/fastStructure --log bool --seed ${SEED} --extra_opts "cv=5 prior=simple full"

*# Record seed value*
echo "Using seed '${SEED}'" **>** ../output/6a_structure_threader/1_all_K1to17_10reps_full/1_bhs_all_table437_5_${i}/1_bhs_all_table437_5_seed_value$i.txt

**done**

**2) All data- used selected Ks in 10 reps (step 1), 100 replicates**

*# Create directory for output*
mkdir -p ../output/6a_structure_threader/2_all_K6to11_100reps

*# Generate random seed*
**for** i in {1..100}
**do**
SEED=$RANDOM

*# Run program and use fivefold validation with 8 threads*
structure_threader run -Klist 6 7 8 9 10 11 -i ../output/1_bhs_all_table437_5.bed -o ../output/6a_structure_threader/2_all_K6to11_100reps/1_bhs_all_table437_5_${i} --pop ../output/6a_structure_threader/1_bhs_all_table437_5.txt -t 8 -fs /usr/local/bin/fastStructure --log bool --seed ${SEED} --extra_opts "cv=5 prior=simple full"

*# Record seed value*
echo "Using seed '${SEED}'" **>** ../output/6a_structure_threader/2_all_K6to11_100reps/1_bhs_all_table437_5_${i}/1_bhs_all_table437_5_seed_value$i.txt

**done**

**Prepare Structure output for Clumpak analysis online**

Create folder for all 100 replicate runs to process

mkdir ../output/6b_structure_results_processing/all_K6to11_100reps

Copy files from individual folders to one folder and rename with source folder name

cd ../output/6b_structure_results_processing/all_K6to11_100reps

shopt -s globstar
**for** file in ../../6a_structure_threader/2_all_K6to11_100reps/**/*.meanQ
**do**
 cp "$file" "${file//\//_}"
**done**

Rename files so that they do not end in periods

cd ../output/6b_structure_results_processing/all_K6to11_100reps

*# Remove first dot*
find -type f -name '.*' -printf '%P\0' **|**
 **while** read -d $'\0' path; **do**
 dir=$(dirname "$path")
 file=$(basename "$path")
 mv "$dir/$file" "$dir/${file#.}"
 **done**

*# Remove second dot*
find -type f -name '.*' -printf '%P\0' **|**
 **while** read -d $'\0' path; **do**
 dir=$(dirname "$path")
 file=$(basename "$path")
 mv "$dir/$file" "$dir/${file#.}"
 **done**

Create directory for clumpak work

mkdir ../output/6b_structure_results_processing/all_K6to11_100reps_for_clumpak

Copy faststructure results

cp -a ../output/6b_structure_results_processing/all_K6to11_100reps/ ../output/6b_structure_results_processing/all_K6to11_100reps_for_clumpak

Remove first four characters of all files to remove special characters

cd ../output/6b_structure_results_processing/all_K6to11_100reps_for_clumpak/all_K6to11_100reps

rename 's/^(.{4})//' *

Zip all the meanQ files into a single zip file

cd ../output/6b_structure_results_processing/all_K6to11_100reps_for_clumpak/all_K6to11_100reps

zip meanq.zip *meanQ

Create population file for clumpak - First column is population number, second column is population name

awk ' { print $4 " " $1 } ' **<**../output/6a_structure_threader/1_bhs_all_table437_5_pops_reordered.csv**>** ../output/6b_structure_results_processing/all_K6to11_100reps_for_clumpak/1_bhs_all_table437_5_pop_clumpak.txt

*# Read in pop file*
herd_list <- **read.table**("../output/6a_structure_threader/1_bhs_all_table437_5.txt", header = FALSE)

*# Read in new order file generated in 6a_structure_threader code*
herd_list_ordered <- **read.table**("../output/6a_structure_threader/1_bhs_all_table437_5_pops_reordered.csv", sep = ",")

*# Label herds with new numbers*
herd_list_clumpak <- **merge**(herd_list, herd_list_ordered, by="V1")

*# Subset file to columns of interest*
herd_list_clumpak <- herd_list_clumpak[, **c**("V4.y", "V1")]

*# Save file*
**write.table**(herd_list_clumpak, file = "../output/6b_structure_results_processing/all_K6to11_100reps_for_clumpak/1_bhs_all_table437_5_pop_clumpak.txt", sep = " ", quote = FALSE, row.names = FALSE, col.names = FALSE)

- Uploaded meanQ and pop_clumpak file to online CLUMPAK server
- Used server default settings: “CLUMPP: default parameters are the LargeKGreedy algorithm, random input order, and 2000 repeats”
- Select ADMIXTURE as file type

**Plot final results from CLUMPAK using pophelper**

- Save CLUMPAK results on local computer and replace CLUMPAK_ONLINE_SERVER_JOB_NUMBER with actual job number

Modify CLUMPAK output file to pophelper format

awk ' { print $1 $5 " " $6 " " $7 " " $8 " " $9 " " $10 " " $11 " " $4} ' **<**../output/6b_structure_results_processing/all_K6to11_100reps_CLUMPAK_output/CLUMPAK_ONLINE_SERVER_JOB_NUMBER/K=6/MajorCluster/CLUMPP.files/ClumppIndFile.output**>** ../output/6b_structure_results_processing/all_K6to11_100reps_CLUMPAK_output/CLUMPAK_ONLINE_SERVER_JOB_NUMBER/K=6/MajorCluster/CLUMPP.files/ClumppIndFile.output_pophelper_format.txt

Read clumpp results back into pophelper

*# Read clumpp results back into pophelper*
clumpp_list_K6 <- **readQ**(files="../output/6b_structure_results_processing/all_K6to11_100reps_CLUMPAK_output/CLUMPAK_ONLINE_SERVER_JOB_NUMBER/K=6/MajorCluster/CLUMPP.files/ClumppIndFile.output_pophelper_format.txt", filetype = "clumpp")

Generate graphics as desired based on pophelper help manual.

**Treemix**

Software requirements:

- PLINK v1.90b4.6 64-bit (15 Aug 2017)
- treemix-1.13
- BITE_1.2.0003 (R package used for bootstrap replicates)
- dplyr (R package)
- stringr (R package)
- ggplot2 (R package)

**LD Decay**

Evaluate LD decay in dataset to set block size in treemix analysis.

- set ld-window to large number
- set ld-window-kb to length of the longest chromosome in kb: OAR1 = 275610

mkdir ../output/7d_treemix_LD_decay

plink --sheep --bfile "../output/7_treemix/bhs_all_table437_5_20plus" --r2 --ld-window-r2 0 --ld-window 999999 --ld-window-kb 275610 --out "../output/7d_treemix_LD_decay/bhs_all_table437_5_20plus"

Create summary file

cat ../output/7d_treemix_LD_decay/bhs_all_table437_5_20plus.ld **|** sed 1,1d **|** awk -F " " 'function abs(v) {return v < 0 ? -v : v}BEGIN{OFS="\t"}{print abs($5-$2),$7}' **|** sort -k1,1n **>** ../output/7d_treemix_LD_decay/bhs_all_table437_5_20plus.ld.summary

Read results into R and process for plotting

**library**(dplyr)
**library**(stringr)
**library**(ggplot2)

dfr <- **read.delim**("../output/7d_treemix_LD_decay/bhs_all_table437_5_20plus.ld.summary",sep="",header=F,check.names=F,stringsAsFactors=F)
**colnames**(dfr) <- **c**("dist","rsq")

*# Group into 10 kb intervals*
dfr**$**distc <- **cut**(dfr**$**dist,breaks=**seq**(from=**min**(dfr**$**dist)**-**1,to=**max**(dfr**$**dist)**+**1,by=100000))

*# Calculate mean and median r2 within blocks*
dfr1 <- dfr **%>%** **group_by**(distc) **%>%** **summarise**(mean=**mean**(rsq),median=**median**(rsq))

*# Get mid-points of distance intervals for plotting*
dfr1 <- dfr1 **%>%** **mutate**(start=**as.integer**(**str_extract**(**str_replace_all**(distc,"[\\(\\)\\[\\]]",""),"^[0-9-e+.]+")), end=**as.integer**(**str_extract**(**str_replace_all**(distc,"[\\(\\)\\[\\]]",""),"[0-9-e+.]+$")), mid=start**+**((end**-**start)**/**2))

*# Plot results*
**ggplot**()**+**
 **geom_point**(data=dfr1,**aes**(x=start,y=mean),size=0.4,colour="grey20")**+**
 **geom_line**(data=dfr1,**aes**(x=start,y=mean),size=0.3,alpha=0.5,colour="grey40")**+**
 **labs**(x="Distance (Megabases)",y=**expression**(LD**~**(r**^**{2})))**+**
 **scale_x_continuous**(breaks=**c**(0,2*****10**^**6,4*****10**^**6,6*****10**^**6,8*****10**^**6),labels=**c**("0","2","4","6","8"))**+**
 **theme_bw**()

**ggplot**()**+**
 **geom_point**(data=dfr1,**aes**(x=start,y=mean),size=0.4,colour="grey20")**+**
 **geom_line**(data=dfr1,**aes**(x=start,y=mean),size=0.3,alpha=0.5,colour="grey40")**+**
 **labs**(x="Distance (Bases)",y=**expression**(LD**~**(r**^**{2})))**+**
 *#scale_x_continuous(breaks=c(0,2*10^6,4*10^6,6*10^6,8*10^6),labels=c("0","2","4","6","8"))+*
 **xlim**(0, 8*****10**^**6)**+**
 **theme_bw**()

**Treemix data analysis**

- Block size 10 used in final analysis

**Convert data to treemix format**

*# Filter out Galton and Highlands to retain samples of >= 20 sample size*
*# Read in sample family data*
family_info <- **read.table**("../output/1_bhs_all_table437_1.fam")

*# Select herds to remove*
family_info_20plus <- **subset**(family_info, family_info**$**V1 **==** "Galton" **|** family_info**$**V1 **==** "Highlands")

*# Write out file*
**write.table**(family_info_20plus[ ,1**:**2], file = "../output/7_treemix/family_info_20plus_for_treemix.txt", row.names=FALSE, col.names = FALSE, sep="\t", quote = FALSE)

*# Pull out the desired samples*
plink --sheep --bfile ../output/1_bhs_all_table437_5 --remove ../output/7_treemix/family_info_20plus_for_treemix.txt --out ../output/7_treemix/bhs_all_table437_5_20plus --make-bed

*# Generate .clust file from family file to define populations*
awk ' { print $1 "\t" $2 "\t" $1} ' **<**../output/7_treemix/bhs_all_table437_5_20plus.fam**>** ../output/7_treemix/bhs_all_table437_5_20plus.clust

*# Convert to frequency format in plink*
plink --sheep --bfile ../output/7_treemix/bhs_all_table437_5_20plus --freq --allow-no-sex --missing --within ../output/7_treemix/bhs_all_table437_5_20plus.clust --out ../output/7_treemix/bhs_all_table437_5_20plus

Extra step- evaluate missing frequencies of SNPs by population after conversion to frequencies

*# Load genotype report file into R*
missing_data_20plus <- **read.table**("../output/7_treemix/bhs_all_table437_5_20plus.lmiss", header = TRUE)

*# Prep plink file for treemix script*
gzip ../output/7_treemix/bhs_all_table437_5_20plus.frq.strat

*# Run treemix conversion python script*
*## Treemix conversion script downloaded from treemix site*
python plink2treemix.py ../output/7_treemix/bhs_all_table437_5_20plus.frq.strat.gz ../output/7_treemix/bhs_all_table437_5_20plus_treemix.frq.gz

**CREATE 50 REPLICATES OF BLOCK SIZE 10- takes about 24 hours to run**

Create directory for output

mkdir ../output/7_treemix/all_data_20plus_evaluate_blocksize/blocksize_10_50reps

Run replicates

*# Generate random seed for 50 replicates*
**for** i in {1..50}
**do**
SEED=$RANDOM

*# Run program*
treemix -i ../output/7_treemix/bhs_all_table437_5_20plus_treemix.frq.gz -root Sierra_Nevada -o ../output/7_treemix/all_data_20plus_evaluate_blocksize/blocksize_10_50reps/bhs_all_table437_5_20plus_treemix_outstem_${i} -global -se -seed ${SEED} -k 10

*# Record seed value*
echo "Using seed '${SEED}'" **>** ../output/7_treemix/all_data_20plus_evaluate_blocksize/blocksize_10_50reps/bhs_all_table437_5_20plus_treemix_seed_value$i.txt

**done**

*# Run replicates with 1-10 migrations*
*# Generate random seed for 50 replicates*
**for** i in {1..50}
**do**
SEED=$RANDOM

*# Record seed value*
echo "Using seed '${SEED}'" **>** ../output/7_treemix/all_data_20plus_evaluate_blocksize/blocksize_10_50reps/bhs_all_table437_5_20plus_treemix_seed_value_migration$i.txt

 *# Run treemix for 1-10 migrations*
 **for** j in {1..10}
 **do**

 *# Run program with migration*
 treemix -i ../output/7_treemix/bhs_all_table437_5_20plus_treemix.frq.gz -root Sierra_Nevada -m ${j} -o ../output/7_treemix/all_data_20plus_evaluate_blocksize/blocksize_10_50reps/bhs_all_table437_5_20plus_treemix_outstem_migration${j}_${i} -global -se -seed ${SEED} -k 10

 **done**
**done**

**Evaluate likelihood and variability by migration value**

*# Loop to pull out file contents*
path = "../output/7_treemix/all_data_20plus_evaluate_blocksize/blocksize_10_50reps/"
out.file<-**list**()
file.names <- **dir**(path, pattern =".llik")
indv_file_names <-**list**()

**setwd**("../output/7_treemix/all_data_20plus_evaluate_blocksize/blocksize_10_50reps/")
**for**(i **in** 1**:length**(file.names)){
 *# Extract file contents*
 file_contents <- **read.table**(file.names[i], header=FALSE, sep=":", stringsAsFactors=FALSE)
 *# Extract file names*
 indv_file_name <- **as.data.frame**(**sapply**(file.names[i], basename))
 *# Create a dataframe of all file names*
 indv_file_names <- **rbind**(indv_file_name, indv_file_name)
 *# Combine file names and output*
 file_contents2 <- **cbind**(file_contents, indv_file_names)
 *# Create a dataframe of all file contents with file names*
 out.file <- **rbind**(out.file, file_contents2)
}

*# Remove starting likelihoods*
likelihood_results_block10 <- out.file[**!grepl**("Starting", out.file**$**V1),]
likelihood_results_block10**$**V1 <- **as.factor**(likelihood_results_block10**$**V1)

MANUSCRIPT PLOT- plot likelihood of 10 block size output vs. number of migration events

**<<<<<<<** HEAD
*# Extract 1 record per migration number as all results are the same*
**=======**
## Extract 1 record per migration number as all results are the same
**>>>>>>>** 3d68fe566a6ba286b555be3acc82cb7cb04540c0
likelihood_results_block10_1entrypermig <- likelihood_results_block10[**!duplicated**(likelihood_results_block10[1]),]

*# Create new column for migration number by extracting numeric value*
**require**(stringr)
likelihood_results_block10_1entrypermig2 <- **transform**(likelihood_results_block10_1entrypermig, Migrations=stringr**::str_extract**(V1, "\\d+"))

*# Make Migrations numeric*
likelihood_results_block10_1entrypermig2**$**Migrations <- **as.numeric**(**levels**(likelihood_results_block10_1entrypermig2**$**Migrations)[likelihood_results_block10_1entrypermig2**$**Migrations])

*# Rename likelihood column name*
**colnames**(likelihood_results_block10_1entrypermig2)[2] <- "Likelihood"

*# Example plot code*
**library**(ggplot2)
likelihood_plot <- **ggplot**(data=likelihood_results_block10_1entrypermig2, **aes**(x = Migrations, y = Likelihood)) **+**
 **geom_point**(size=1) **+**
 **geom_text**(**aes**(label=''), hjust=0, vjust=0, show.legend = FALSE) **+**
 **scale_y_continuous**(breaks=**pretty**(likelihood_results_block10_1entrypermig2**$**Likelihood),
 limits = **range**(**pretty**(likelihood_results_block10_1entrypermig2**$**Likelihood))) **+**
 **scale_x_continuous**(breaks = **c**(0,1,2,3,4,5,6,7,8,9,10))**+**
 *#scale_x_continuous(breaks=pretty(likelihood_results_block10_1entrypermig2$Migrations),*
 *# limits = range(pretty(likelihood_results_block10_1entrypermig2$Migrations))) +*
 **theme**(axis.title.x = **element_text**(face="bold", colour="black"),
 axis.text.x = **element_text**(face="bold", colour="black"),
 axis.title.y = **element_text**(face="bold", colour="black", vjust=2),
 axis.text.y = **element_text**(face="bold", colour="black", vjust=0.5),
 axis.line = **element_line**(),
 panel.background=**element_blank**(),
 panel.border=**element_blank**(),
 panel.grid.major=**element_blank**(),
 panel.grid.minor=**element_blank**(),
 plot.background=**element_blank**(),
 plot.title = **element_text**( face="bold"),
 legend.title = **element_text**(colour="black", face="bold"),
 legend.text = **element_text**(colour="black"),
 legend.key = **element_blank**(),
 legend.key.height = **unit**(1,"line"),
 legend.background = **element_rect**(colour = "black")) **+**
 **xlab**("\nNumber of Migration Events") **+**
 **ylab**("Likelihood\n")
**print**(likelihood_plot)

*Calculate variability explained by model and add to likelihood table*

Example code to extract variability explained by the model for a run:

*# Tell R source code location*
**source**("~/Downloads/treemix-1.13/src/plotting_funcs.R")

*#Pull out file contents*
path = "../output/7_treemix/all_data_20plus_evaluate_blocksize/blocksize_10_50reps/"
file_names <- **dir**(path, pattern =".modelcov.gz")

*#Remove extension so get_f can read file names*
file_names2 <- **sub**(".modelcov.gz", "", file_names)

*# Run function on all files in folder*
**setwd**("../output/7_treemix/all_data_20plus_evaluate_blocksize/blocksize_10_50reps/")
variability_results_block10 <- **sapply**(file_names2, get_f)

variability_results_block10 <- **as.data.frame**(variability_results_block10)

MANUSCRIPT PLOT- plot variability of 10 block size output vs. number of migration events

**<<<<<<<** HEAD
*# Extract 1 record per migration number as all results are the same*
**=======**
## Extract 1 record per migration number as all results are the same
**>>>>>>>** 3d68fe566a6ba286b555be3acc82cb7cb04540c0
variability_results_block10**$**names <- **rownames**(variability_results_block10)
variability_results_block10_1entrypermig <- **as.data.frame**(variability_results_block10[**!duplicated**(variability_results_block10[1]),])

*# Create new column for migration number by extracting numeric value*
**require**(stringr)
variability_results_block10_1entrypermig2 <- **transform**(variability_results_block10_1entrypermig, Migrations=stringr**::str_extract**(names, "migration(\\d+)"))
variability_results_block10_1entrypermig2**$**Migrations <- **as.character**(variability_results_block10_1entrypermig2**$**Migrations)
variability_results_block10_1entrypermig2 <- **transform**(variability_results_block10_1entrypermig2, Migrations=stringr**::str_extract**(Migrations, "\\d+"))
variability_results_block10_1entrypermig2**$**Migrations <- **ifelse**(**is.na**(variability_results_block10_1entrypermig2**$**Migrations), "0", variability_results_block10_1entrypermig2**$**Migrations)

*# Make Migrations numeric*
variability_results_block10_1entrypermig2**$**Migrations <- **as.numeric**(**as.character**(variability_results_block10_1entrypermig2**$**Migrations))

*# Rename variability column name*
**colnames**(variability_results_block10_1entrypermig2)[1] <- "Variability"

*# Example plot code*
**library**(ggplot2)
variability_plot <- **ggplot**(data=variability_results_block10_1entrypermig2, **aes**(x = Migrations, y = Variability)) **+**
 **geom_point**(size=1) **+**
 **geom_text**(**aes**(label=''), hjust=0, vjust=0, show.legend = FALSE) **+**
 **scale_y_continuous**(breaks=**pretty**(variability_results_block10_1entrypermig2**$**Variability),
 limits = **range**(**pretty**(variability_results_block10_1entrypermig2**$**Variability))) **+**
 **scale_x_continuous**(breaks=**c**(0,1,2,3,4,5,6,7,8,9,10)) **+**
 **theme**(axis.title.x = **element_text**(face="bold", colour="black"),
 axis.text.x = **element_text**(face="bold", colour="black"),
 axis.title.y = **element_text**(face="bold", colour="black", vjust=2),
 axis.text.y = **element_text**(face="bold", colour="black", vjust=0.5),
 axis.line = **element_line**(),
 panel.background=**element_blank**(),
 panel.border=**element_blank**(),
 panel.grid.major=**element_blank**(),
 panel.grid.minor=**element_blank**(),
 plot.background=**element_blank**(),
 plot.title = **element_text**( face="bold"),
 legend.title = **element_text**(colour="black", face="bold"),
 legend.text = **element_text**(colour="black"),
 legend.key = **element_blank**(),
 legend.key.height = **unit**(1,"line"),
 legend.background = **element_rect**(colour = "black")) **+**
 **xlab**("\nNumber of Migration Events") **+**
 **ylab**("Variability Explained\n")
**print**(variability_plot)

**Evaluate residuals**

*# Create population file automatically*

*# Generate .phe file from family file to define populations*
awk ' { print $1 "\t" $2 "\t" $1} ' **<**../output/7_treemix/bhs_all_table437_5_20plus.fam**>** ../output/7_treemix/bhs_all_table437_5_20plus.phe

awk '!($1 in a){a[$1];print}' **<**../output/7_treemix/bhs_all_table437_5_20plus.phe**>** ../output/7_treemix/bhs_all_table437_5_20plus_treemix_poporder.txt

awk ' { print $1 } ' **<**../output/7_treemix/bhs_all_table437_5_20plus_treemix_poporder.txt**>** ../output/7_treemix/bhs_all_table437_5_20plus_treemix_poporder2.txt

MANUSCRIPT PLOT- TREEMIX RESIDUALS

*# Tell R source code location for drawing tree*
**source**("~/Downloads/treemix-1.13/src/plotting_funcs.R")

*# Create population order file- designate order of populations manually if needed*

*# Create pdf*
**pdf**("../manuscript/S4_treemix_residuals_bhs_all_table437_5_20plus_treemix_outstem_migration4_1_blocksize10.pdf", width=6, height=5)
mar.default <- **c**(5,4,4,2) **+** 0.1
**par**(mar = mar.default **+** **c**(4, 4, 0, 0))
**plot_resid**("../output/7_treemix/all_data_20plus_evaluate_blocksize/blocksize_10/bhs_all_table437_5_20plus_treemix_outstem_migration4_1", pop_order = "../output/7_treemix/bhs_all_table437_5_20plus_treemix_poporder2.txt")
**dev.off**()

*# Create png- resolution not defined*
**png**("../manuscript/S4_treemix_residuals_bhs_all_table437_5_20plus_treemix_outstem_migration4_1_blocksize10.png")
mar.default <- **c**(5,4,4,2) **+** 0.1
**par**(mar = mar.default **+** **c**(4, 4, 0, 0))
**plot_resid**("../output/7_treemix/all_data_20plus_evaluate_blocksize/blocksize_10/bhs_all_table437_5_20plus_treemix_outstem_migration4_1", pop_order = "../output/7_treemix/bhs_all_table437_5_20plus_treemix_poporder2.txt")
**dev.off**()

**Use BITE to create bootstrap replicates in Treemix and plot results**

*# ANALYSIS: 4 migrations, Block size 10*

*# 1) Set up directories*
*# Create output folder for run*
mkdir ../output/7a_treemix_bite/bootstrap_mig4_blocksize10

*# Copy bootstrap script to output folder*
cp ./treemix_scripts/Treemix_bootstrap.sh ../output/7a_treemix_bite/bootstrap_mig4_blocksize10/Treemix_bootstrap.sh

*# Copy input file to output folder*
cp ../output/7_treemix/bhs_all_table437_5_20plus_treemix.frq.gz ../output/7a_treemix_bite/bootstrap_mig4_blocksize10/

*# 2) Create bootstrap replicates*
cd ../output/7a_treemix_bite/bootstrap_mig4_blocksize10/
*# Run bootstrap analysis using 4 migrations, 1000 replicates*
./Treemix_bootstrap.sh bhs_all_table437_5_20plus_treemix.frq.gz 4 8 10 Sierra_Nevada 1000 ~/Downloads/phylip-3.697/exe/consense 1_bhs_all_table437_5_treemix_outstem_bootstrap_mig4_blocksize10

**Bootstrap replicate plot: 4 migrations, Block size 10**

- newick file indicates bootstrap support for all nodes

*# Set working directory of output*
**setwd**("../output/7a_treemix_bite/bootstrap_mig4_blocksize10/")

*# Plot the Treemix tree with migrations and, on branches, the bootstrap values*
## Identify output file names twice
## Set number of bootstrap replicates used
**pdf**("Treemix_plot_1_bhs_all_table437_5_treemix_outstem_bootstrap_bootstrap_mig4_blocksize10.pdf", 10, 7)
**treemix.bootstrap**(in.file = "1_bhs_all_table437_5_treemix_outstem_bootstrap_mig4_blocksize10",
 out.file = "outfile",
 phylip.file = "1_bhs_all_table437_5_treemix_outstem_bootstrap_mig4_blocksize10_outtree.newick",
 *# pop.color.file = "PopOrdCol.txt",*
 nboot = 1000, *# Change to number of bootstraps used*
 *# cex=0.5, xmin = -0.005, disp = 0.001 # Change settings for graph appearance*
 )
**dev.off**()

**Three-population test**

threepop -i ../output/7_treemix/bhs_all_table437_5_20plus_treemix.frq.gz -k 10 **>** ../output/7_treemix/threepop/threepop_blocksize_10.txt
